# The influence law of eccentric load on the performance of yielding bolt

**DOI:** 10.1098/rsos.200227

**Published:** 2020-07-01

**Authors:** Yang Tai, Hongchun Xia, Shaoping Huang, Jie Meng, Wei Li

**Affiliations:** 1 School of Mines, China University of Mining and Technology, Xuzhou 221116, People's Republic of China; 2 College of Civil Engineering and Architecture, Dalian University, Liaoning, Dalian 116622, People's Republic of China; 3 Faculty of Engineering, China University of Geosciences, Wuhan, Hubei 430074, People's Republic of China; 4 Department of Civil Engineering, University of Ottawa, 161 Colonel By, Ottawa, Ontario, Canada K1N 6N5

**Keywords:** yielding bolt, eccentric load, performance, numerical simulation

## Abstract

In order to adapt to the high stress and avoid the large deformation in roadways, the pre-stressed yielding bolt has been developed. Prior to the installation of the pre-stressed yielding bolt, boreholes need to be drilled. However, not all boreholes are perpendicular to the surface of the roadway, and the non-perpendicular holes make the pre-stressed yielding bolt exposed to eccentric loads. In order to reveal the influence of the eccentric load on the performance of the pre-stressed yielding bolt, some numerical simulations were carried out in this study. The influence of the eccentric load on the displacement–load relations, utilization rate of the yielding pipe, the plastic strains of the bolt components as well as the evolution of the absorptive capacity of the yielding pipe were analysed. The results are as follows: (i) eccentric loads affected the utilization rate of the yielding pipe, plastic strains of bolt components and the absorptive capacity was quite great when displacement was less than 2 mm, while these impacts could be neglected when displacement is greater than 2 mm; (ii) as the eccentric load increased, the yielding point and its corresponding displacement increased linearly while the yielding magnitude decreased linearly; and (iii) the eccentric load could be adjusted to control the yielding point and magnitude in order to meet the roadway support's requirement for the yielding bolt.

## 1. Introduction

**Figure 1.**
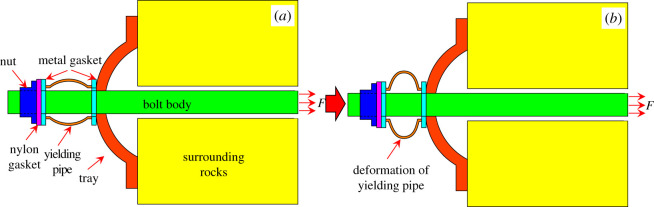
The structure of the yielding bolt.

Rock bolting support is one of the most common methods used in coal mines for its good controlling effect, low cost and simple operation [1,2]. As the mining depth of coal mines increases, however, the roadways are subjected to a high stress which could potentially lead to a large deformation [3]. Accordingly, it is necessary to optimize the structures of normal rock bolts to meet the support requirements in this condition [4]. To date, various types of yielding bolts have been designed, among which, the pre-stressed yielding bolt is most popular one (developed by the Jennmar Corporation, as shown in figure 1) [5]. It consists of a nut, a plate and high-intensity bolt body, and a yielding pipe made of seamless steel pipe. Ideally in this structure, the excessive deformation energy could be released through the deformation of the yielding pipe [6,7] (as shown in (figure 1*b*)). And when that occurs, the constraint force of the bolt would increase, which would reinforce the steadiness of surrounding rocks. As a result, the stability of the roadway would be better ensured [8].

**Figure 2.**
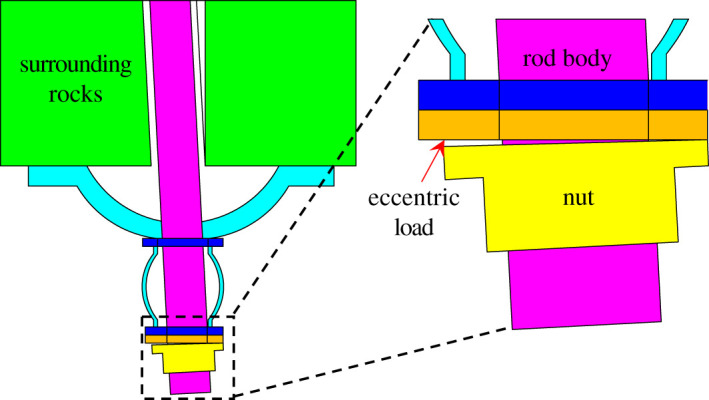
The eccentric load for yielding bolt.

In reality, however, before installation of the yielding bolt, a hole needs to be drilled with professional equipment. Due to influences of uncontrollable factors such as drill pattern design, equipment accuracy and human error, borehole deviation often occurs during the drilling process, as shown in figure 2. Consequently, the eccentric load will happen to the pre-stressed yielding bolt.

Numerous studies have been made on the yielding bolt, mainly focusing on three aspects. Beginning with the evaluation of the yielding effects, Xiang *et al*. [9] established a mechanical model of the yielding bolt to evaluate the yielding effect. He used the finite-element shear strength reduction method to analyse and evaluate the support effects of the high-intensity yielding bolt. Zhu *et al*. [10] built a constitutive model of the yielding anchor, and used the FLAC^3D^ software to analyse the slope reinforcement effects of the anchor. Yang [11] introduced the structure and support procedure of the large-scale yielding bolt, analysed its performance at the stages of elastic deformation, sliding and yielding with the ANSYS software, and evaluated the yielding results. Lian & Wang [12] built a three-dimensional mechanical model for the shear failure between the pre-stressed yielding bolt and the rock, and developed a corresponding calculation method. Secondly, with respect to key parameters selection of the yielding bolt, Shan *et al*. [13] divided the whole tensile process of the yielding bolt into the elastic stage, the yielding stage and the plastic hardening stage. Based on the energy constitutive equations at different stages, the ultimate load calculation formula was given. Yang [14] discussed various yielding bolts' basic mechanical parameters such as the yielding point, yielding load stability and the maximum yielding amount, which could provide basis for the selection of the yielding bolt. Thirdly, regarding the coupling relationships between the yielding bolt and the surrounding rock, Guo *et al*. [15] introduced a new type of pressure-dispersive yielding bolt. He analysed the mechanism and mechanical behaviours and the coupling relationships between the yielding bolt and the surrounding rock. Zhang *et al*. [16] systematically analysed variations of surrounding rock stresses, displacements and axial forces, before and after the installation of the yielding bolt. In terms of failure models, the yielding bolt could be subjected to two kinds of movements—either opening in the direction perpendicular to the plane or shearing in the plane [7,17]. However, the failure of anchoring systems is mainly attributed to the shear loading. Indeed, many previous studies have performed experiments to study factors that affect the shear behaviour of bolt, including bolt type, bolt diameter, bolt surface profile and bolt material [18–20].

With the above being said, however, the influence of eccentric load on the performance of the yielding bolt has not been studied. Therefore, this study established a numerical model of the yielding bolt, through which the effects of eccentric loads on the performance of the yielding bolt were explored and summarized. The engineering design was carried out to verify the accuracy of the model.

## 2. Numerical calculation method

### 2.1. Calculation method

#### 2.1.1. Method of solving nonlinear equations

**Figure 3.**
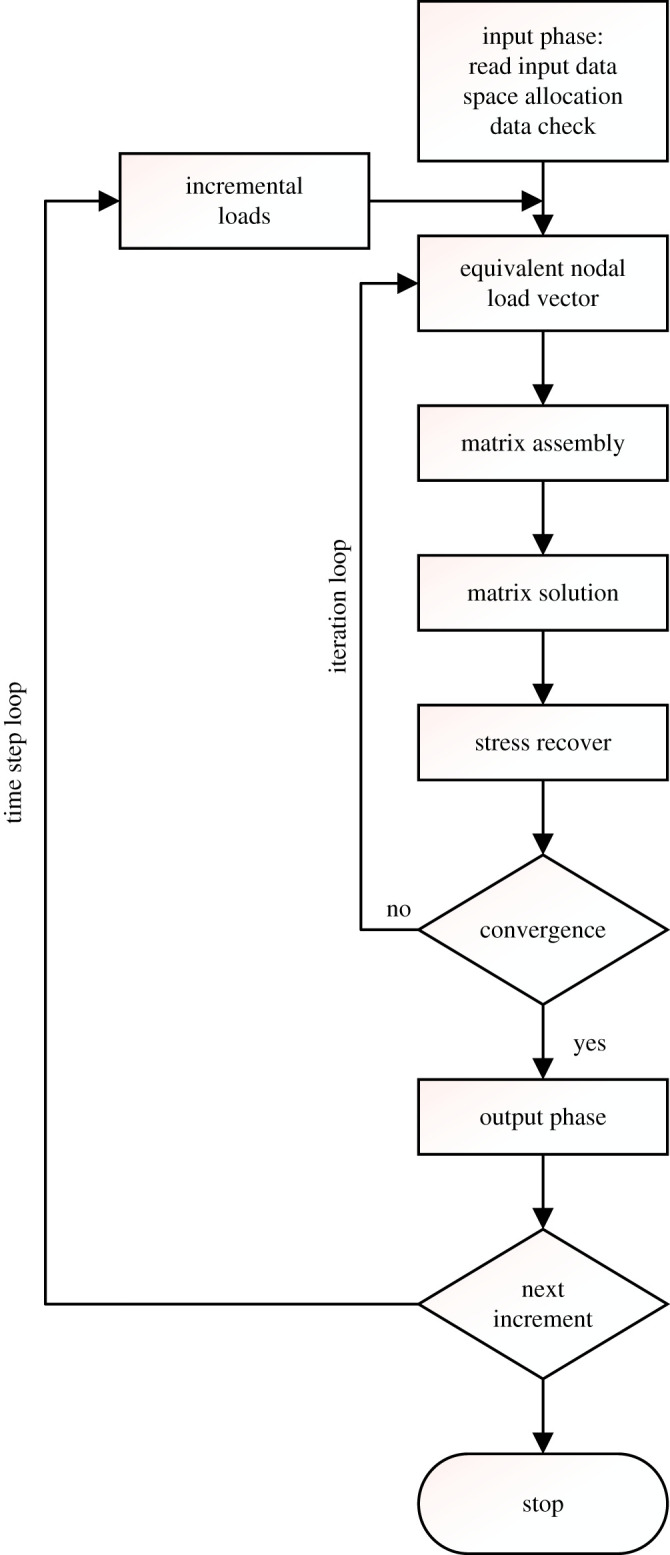
Diagram of solutions.

During the yielding process of the yielding bolt, multiple nonlinearities are involved, such as the geometric nonlinearity, material nonlinearity and contact nonlinearity. In addition, due to the complex bolt structure, the traditional theoretical calculations could not fully reveal the influence of eccentric load on the performance of the yielding bolt. Thus, the numerical simulation was adopted to solve the problem, whose basic flow is shown in figure 3.

It could be seen that matrix solution involves many nonlinear equations; therefore, solving nonlinear equations is the most critical part for the numerical calculation.

A nonlinear system is expressed as

2.1 $$K^{T}\Delta u=F-R=r,$$

where *K* is the elastic stiffness matrix, *K*^T^ is the tangent stiffness matrix and Δ*u* the displacement vector. *F* is the applied load vector, *R* is the internal nodal-load vector and *r* the residual.

The iteration is a common method to solve nonlinear problems. The objective of the iterative method is to minimize the complexity of the function.

In the simplest method

2.2 $$u_{k+1}=u_{k}+\alpha_{k}r_{k},$$

where *u_k_* and *u_k_*_+1_ are the displacement vectors at the *k*th iteration and (*k* + 1)th iteration, while *r_k_* is the residual at the *k*th iteration

2.3 $$\alpha_{k}=\frac{r_{k}^{T}r_{k}}{r_{k}^{T}Kr_{k}}.$$

However, the gradient directions show little differences, which results in a poor convergence.

To mitigate the problem, an improved method, the *conjugate gradient method*, is introduced:

2.4 $$u_{k+1}=u_{k}+\alpha_{k}p_{k}$$

and

2.5 $$\alpha_{k}=\frac{\;p_{k}^{T}r_{k-1}}{\;p_{k}^{T}Kr_{k}}.$$

The essential part is to choose *p_k_* to be *K* conjugate to *p*_1_, *p*_2_, …, *p*_k-1_, which is why the method is named as *conjugate gradient methods*. The elegance of this method is that the solution may be obtained through a series of matrix multiplications and the stiffness matrix never needs to be decomposed.

#### 2.1.2. The iterative method

The Newton–Raphson method is used in the numerical simulation. During the calculation, equilibrium needs to be satisfied in structural analysis. Considering the following set of equations

2.6 $$K^{T}(u)\delta u=F-R(u),$$

where *u* is the nodal displacement vector, *F* is the applied load vector, *R* the internal nodal-load vector and *K*^T^ the tangent stiffness matrix. The applied load vector is obtained from internal stresses

2.7 $$R=\sum_{\mathrm{elem}}\int_{V}\beta^{T}\sigma \;\text{d}v.$$

In this set of equations, both *R* and *K*^T^ are functions of *u*. In many cases, *F* is also a function of *u* (for example, if *F* follows pressure loads, the nodal-load vector is a function of the structure orientation). The equations suggest that full Newton–Raphson method is appropriate.

Suppose that the last obtained approximate solution is termed *δu^i^*, where *i* indicates the iteration number. Equation (6) can then be written as

2.8 $$K^{T}(u_{n+1}^{i-1})\delta u^{i}=F-R(u_{n+1}^{i-1}).$$

This equation could be used to solve for *δu^i^* and the next appropriate solution is obtained by

2.9 $$\begin{array}{rl} \Delta u^{i}= & \Delta u^{i-1}+\delta u^{i}\\ \;\text{and}\;u_{n+1}^{i}= & u_{n+1}^{i-1}+\delta u^{i}. \end{array}\}$$

After the above equation is completed, one iteration is also finished. Then the process can be repeated. The subscript *n* denotes the increment number. In general, the subscript *n* + 1 is dropped with all quantities referring to the current state.

### 2.2. Contact method

There are multiple contacts involved in the numerical simulation, such as contacts among the nylon gasket, metal gasket, the yielding pipe and the plate. In addition, self-contact may occur to the yielding pipe during the yielding process. According to Newton's third law, the contact surface should satisfy the following equations

2.10 $$\begin{array}{c} t_{N}^{A}+t_{N}^{B}=0\;\\ t_{T}^{A}+t_{T}^{B}=0,\; \end{array}\}$$

where $t_{N}^{A}$ and $t_{N}^{B}$ are normal contact forces of *A* and *B*, respectively; $t_{T}^{A}$ and $t_{T}^{B}$ are tangential contact forces of *A* and *B*, respectively. The contact is achieved by the symmetric penalty function method of finite elements, and the main calculation steps are described below.

**Figure 4.**
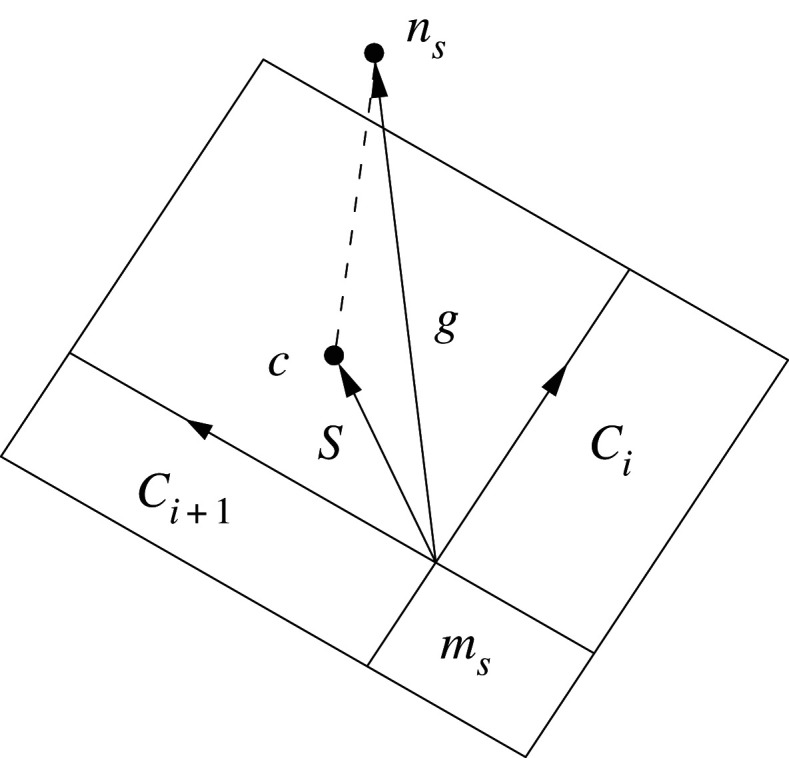
All master surfaces related with *m_s_*_._

I. As shown in figure 4, the first step is to search the closest host node *m_s_* for any slave node *n_s_*.

II. Check all master surfaces related to the master node *m_s_* and determine the possible contact with master surfaces when the slave node *n_s_* penetrates the main elements. If the master node *m_s_* does not coincide with the slave node, and formula (2.11) is satisfied, the node *n_s_* will contact the main element surface *S_i_*

2.11 $$\begin{array}{c} (C_{i}\times S_{i})\cdot (C_{i}\times C_{i+1})>0\;\\ (C_{i}\times S_{i})\cdot (S_{i}\times C_{i+1})>0.\; \end{array}\}$$

In the above formula, *C_i_* and *C_i_*_+__1_ are two edge vectors at the node *m_s_* in the main element surface. Vector *S* is the projection of vector *g* on the main element surface, and *g* is the vector pointing from the master node *m_s_* to the slave node *n_s_*

2.12 $$S=g-(gm)m,m=\frac{C_{i}C_{i+1}}{\left|C_{i}C_{i+1}\right|}.$$

If node *n_s_* is in or near the intersection between two elements’ surfaces, the above equation may be uncertain, and the following formula could be used

2.13 $$S=\text{max}\left(\frac{gC_{i}}{\left|C_{i}\right|}\right)i=1,\;2,\;\ldots$$

III. Determine the position of the contact point *c* of the slave node *n_s_* in the main element's surface. The position vector *p* at any point in the main element surface could be expressed as follows:

2.14 $$p=f_{1}(\xi ,\eta )i_{1}+f_{2}(\xi ,\eta )i_{2}+f_{3}(\xi ,\eta )i_{3},$$

where, $f_{i}(\xi ,\eta )=\sum_{\;j=1}^{4}\phi_{j}(\xi ,\eta )x_{i}^{j}$,$\phi_{j}(\xi ,\eta )=(1+\xi_{j}\xi )(1+\eta_{j}\eta )/4$ and $x_{i}^{j}$ is the *x_i_* coordinate value of the *j*th node of the element; $i_{1},i_{2},i_{3}$ are position vectors of $x_{1},x_{2},x_{3}$ with the coordinates. The position of the contact point $c(\xi_{c},\eta_{c})$ is the solution of the following formulae

2.15 $$\begin{array}{c} \frac{\partial p}{\partial \xi }(\xi_{c},\eta_{c})\cdot [t-p(\xi_{c},\eta_{c})]=0\;\\ \frac{\partial p}{\partial \eta }(\xi_{c},\eta_{c})\cdot [t-p(\xi_{c},\eta_{c})]=0.\; \end{array}\}$$

IV. Check whether the slave node penetrates the main element's surface.

If $l=n_{i}[t-p(\xi_{c},\eta_{c})]<0,$ it means that the slave node *n_s_* penetrates the main element surface with the contact point $c(\xi_{c},\eta_{c}),$ where *n_i_* is the unit outer normal vector of the main element surface at the contact point

2.16 $$n_{i}=\frac{\partial p}{\partial \xi }(\xi_{c},\eta_{c})\times \frac{\partial p}{\partial \eta }(\xi_{c},\eta_{c})/\left|\frac{\partial p}{\partial \xi }(\xi_{c},\eta_{c})\times \frac{\partial p}{\partial \eta }(\xi_{c},\eta_{c})\right|.$$

**Figure 5.**
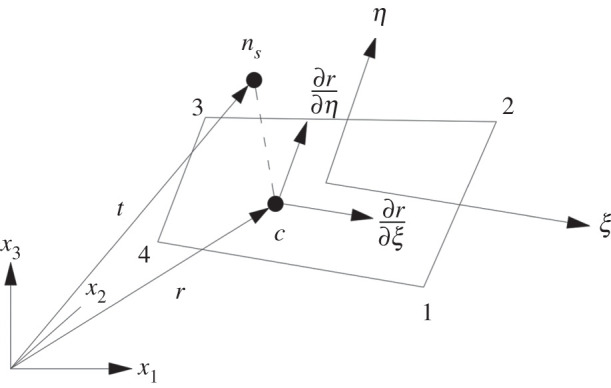
The relationship between the slave node and the master surface.

If *l* ≥ 0, it means that the slave node *n_i_* does not penetrate the main element's surface. Therefore, there is no contact or collision between two objects. The process is completed at the node *n_i_* without any treatment. The search for the relationship between the slave node and the main element's surface will be started from the next slave node *n_i_*_+1_, as shown in figure 5.

V. If the slave node penetrates the main element surface, the normal contact force shall be applied between the slave node *n_s_* and the contact point *c*

2.17 $$f_{s}=-k_{i}n_{i},$$

where *k_i_* is the stiffness factor of the main element surface and meets the following formula

2.18 $$k_{i}=\frac{\;fK_{i}A_{i}^{2}}{V_{i}},$$

where *K_i_* is the bulk modulus of the contact element; *A*_i_ is the area of the main element surface; *V*_i_ is the volume of the main element; *f* is a scale factor of the contact stiffness and 0.10 is set as the default value. In the case of excessive penetration during calculation, keyword controlling parameters could be used to amplify penalty factors. If *f* > 0.4, the calculation may become unstable, unless the time step is reduced.

To apply contact force vector *f_s_* in slave node *n_s_*, according to Newton's third law, a reactive force −*f_s_* acts on the contact point *c* in the main element surface. The contact force at point *c* could be assigned equivalently to the nodes on the main element according to the following formula:

2.19 $$f_{\;jm}=-\phi_{j}(\xi_{c},\eta_{c})f_{s},\;j=1,\;2,\;3,\;4,$$

where *f_jm_* is contact force at the nodes, $\phi_{j}(\xi_{c},\eta_{c})$ is a two-dimensional shape function in the main element surface and $\sum_{\;j=1}^{4}\phi_{j}(\xi_{c},\eta_{c})=1$ at the contact point *c*.

VI. Calculate the tangential contact friction force.

If the normal force of the slave node *n_s_* is *f_s_*, the maximum friction will be $F_{Y}=\mu |\;f_{s}|$ and *μ* is the friction coefficient. Suppose that the contact friction force of *n_s_* is *F^n^* at the last time (*t_n_*), the friction F* at the present time (*t_n_*_+1_) may be $F^{*}=F^{n}-k\Delta \alpha$, where *k* is interface stiffness and $\Delta \alpha =p^{n+1}(\xi_{c}^{n+1},\eta_{c}^{n+1})-p^{n+1}(\xi_{c}^{n},\eta_{c}^{n})$. The contact friction force at the present time could be fixed by the following formula:

2.20 $$F^{n+1}=\{\begin{array}{cc} F^{*}\; & \text{if}\;|F^{*}|\leq F_{Y}\\ F_{Y}F^{*}/|F^{*}| & if\;|F^{*}|>F_{Y} \end{array}.$$

## 3. Numerical simulation construction of the yielding bolt

### 3.1. Parameters of the yielding bolt

There are many types of yielding bolts. Of them, the most common type is MSGLW series yielding bolt, and the specific mechanical parameters of the bolt body are shown in table 1.

**Table 1.** Mechanical parameters of the rod body.

name and spec.	yield strength (MPa)	tensile strength (MPa)	elongation (%)
MSGLW-500/20, 22(RY)	≥500	≥630	≥15
MSGLW-600/20, 22(RY)	≥600	≥750	

Tables 1 and 2 also show basic parameters of the MSGLW series yielding pipe, one of the core components of the yielding bolt. Since this paper focuses on the effects of eccentric load on the performance of the yielding bolt, the numerical simulation analysis was carried out by taking the yielding bolt in MSGLW-500/20(RY) series as an example. The yield strength of the material is 500 MPa and the bolt diameter is 20 mm. The yielding point appears when the load reaches 160 kN. The maximum yielding amount is 25 mm, and the yielding pipe height is 40 mm.

**Table 2.** Basic parameters of the yielding pipe.

yield strength of yielding pipe (MPa)	diameter (mm)	yielding point (kN)	yielding amount (mm)	inner diameter of yielding pipe (mm)	outer diameter of yielding pipe (mm)	yielding pipe height (mm)
500	20	160 ± 20	25 ± 3	25	40.5	40 ± 2
	22	180 ± 20		25	40.5	
600	20	180 ± 20		25	40.5	
	22	220 ± 20		25	40.5	

### 3.2. Numerical model

In total, there are seven steps in the numerical modelling, including geometric modelling, material selection, mesh generation, contact definition, boundary conditions application, solving and post-processing [21,22]. During the geometric modelling, the three-dimensional software SolidWorks was adopted. Commands, such as rotate and cut, were used to create the gasket, the yielding pipe and the plate. Their corresponding spatial relations were defined in the component module [23]. Due to its symmetry, only half of the model needs to be created, which reduces the calculated amount [24,25]. For the second step, material selection, a bilinear isotropic elastoplastic constitutive model was selected for the metal gasket, plate and the yielding pipe, while the constitutive equation of nylon material would base on the strain energy function [26,27]. To define the contact relations, there were five pairs of contact relations among the bolt's components, and two pairs of contact relations within the components themselves [28]. In terms of mesh generation, the mesh size was set based on the geometry's curvatures and contact relations. Generally, the mesh-density is higher when the curvature is larger. Plus, the mesh size of the slave surface was smaller than that of the master surface [29] in the contact relation. According to these two principles, the mesh size was set between 0.6 and 3.0 mm. A total of 45 430 tetrahedral elements and 1296 quadrilateral elements were generated [30]. For boundary conditions, the symmetry constraints were imposed on the symmetry surfaces, and the bottom of the plate was fixed [31]. In solving the model, the generated mesh model was imported into Nastran Solver, and the advanced nonlinear solver was applied. Finally, the results were imported into Patran to extract values like various stresses, plastic strains.

**Figure 6.**
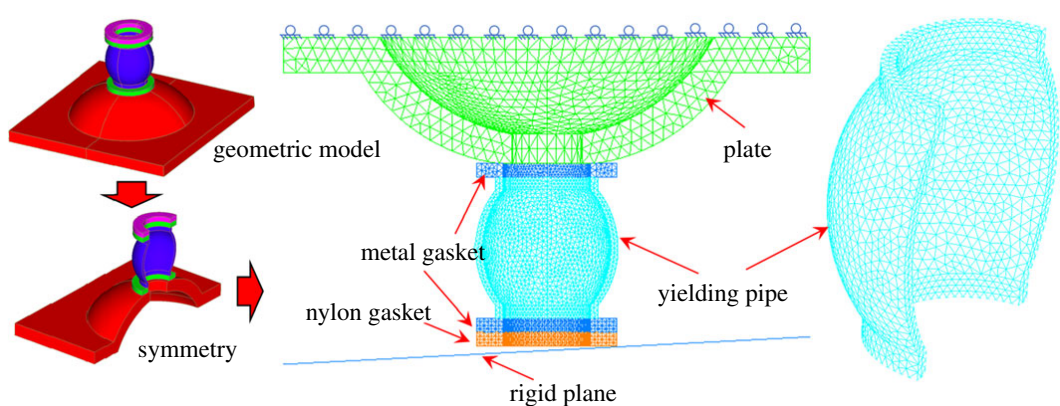
The sketch diagram of the numerical model.

In order to simulate the load that acted on the yielding pipe from the tension of the anchor, the rigid plane was added in the numerical modelling, and its reference points were defined [32]. By adjusting the rigid plane's angle, as shown in figure 6, the influence of eccentric load on the pre-stressed yielding bolt's performance were analysed. Given the hole deviation should be generally between 0° and 5°, the dip of the rigid plane should also be controlled within 5°.

## 4. Analysis of the influence of eccentric load on the yielding pipe's performance

The yielding pipe's performance could be evaluated through its displacement–load relations, efficiency, plastic strain of its components and the absorbed energy. This study also mainly focuses on these four indicators.

### 4.1. The influence of eccentric load on displacement–load relations

**Figure 7.**
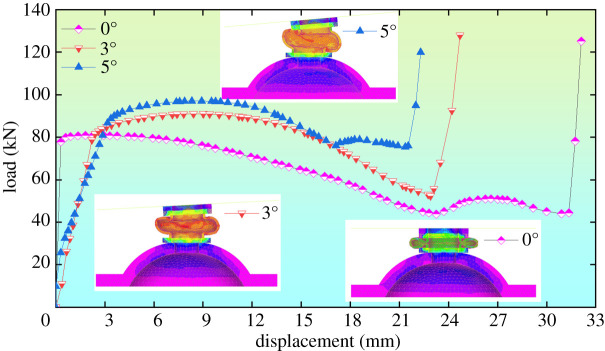
The displacement–load relations under various eccentric load.

The displacement–load relations are used to acquire the yielding bolt's yielding point and amounts, two parameters that could be used to choose proper pre-stressed yielding bolts. The displacement–load relations could be extracted by the post-processing function of the Patran software, as shown in figure 7.

**Figure 8.**
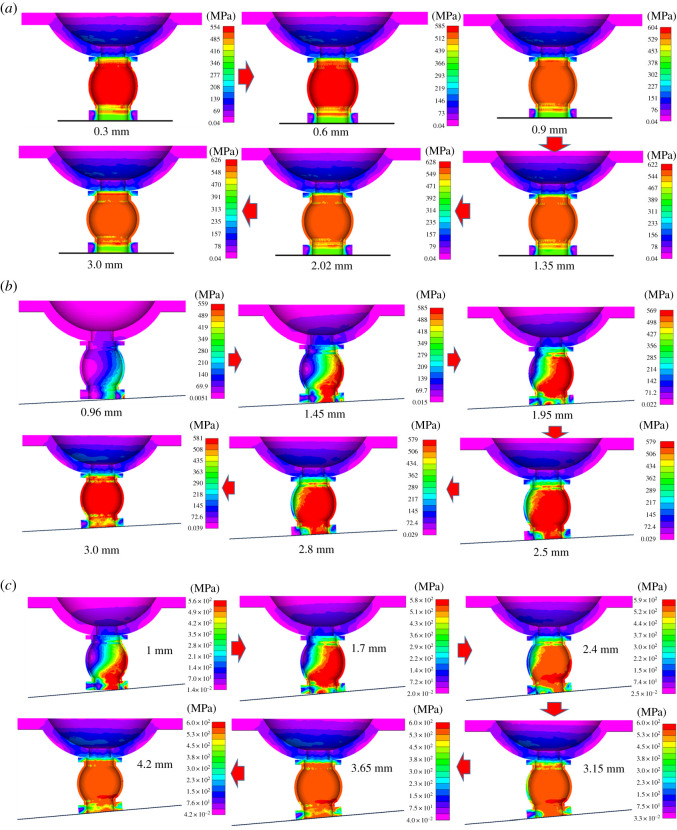
The von Mises stress distribution characteristics of yielding bolt components.

Figure 7 indicates that:

(1) When the eccentric loads are 0°, 3° and 5° off the centre line, the yielding points of half of the yielding pipe are 80.9, 90.1 and 97.0 kN, respectively. The corresponding yielding points of the whole pipe are 161.8, 180.2 and 194 kN, and displacement of the yielding points are 1.42, 7.81 and 8.96 mm, respectively.
(2) As the eccentric load increases, the pipe's yielding point and the corresponding displacement also increase. This could be explained by the following figures. In figure 8*a*, when the eccentric load is 0°, extrusion from the rigid plane makes the entire stress distribution of the pipe even, and there are loads on the integral material. In figure 8*b,c*, when the eccentric loads are 3° and 5°, the pipe's stress spreads from one side to the whole, and the displacement of the yielding point increases. That is due to the change of the pipe's yielding route caused by the eccentric load leading.
(3) When the eccentric loads are 0°, 3° and 5°, the pipe's yielding amounts are 31.2, 23.5 and 21 mm, respectively. The yielding amount increases with the eccentric load.

In order to further explore the relations among eccentric loads, the yielding points, their corresponding displacements and the yielding amounts were all recorded within an eccentric load range of 0°–5°. Their functional relationships are listed in table 3.

**Table 3.** Influence of eccentric loads on key parameters of the yielding bolt.

eccentric load *θ* (°)	yielding point *F*_max_ (kN)	yielding point's displacement *L*_1_ (mm)	yielding amount *L*_2_ (mm)
0	161.8	1.4	31.2
1	166.2	4.2	28.0
2	174.4	6.0	25.3
3	180.2	7.8	23.5
4	190.0	8.2	22.0
5	194.0	8.96	21.0
fitting relation	*F*_max_ = 6.8058*θ* + 160.752	*L*_1_ = 1.4717*θ* + 2.419	*L*_2_ = −2.0229*θ* + 30.224
correlation coefficient	*R*² = 0.9895	*R*² = 0.9237	*R*² = 0.9597

### 4.2. The utilization rate of the yielding pipe

The utilization rate can be defined as to what extent the yielding pipe could bear the loads evenly. In other words, when the yielding pipe is bulking evenly, the utilization rate is marked as high; when only local deformation or even failure happens, the utilization rate is scored as low. The loads could be reflected through the pipe's von Mises stress distribution characteristics. According to §4.1, the von Mises stress concentration occurred at the initial stage of displacement under eccentric load, while the even distribution of the von Mises stress occurred at the later stage of displacement. Therefore, the von Mises stresses were extracted when the deformation amounts were 2 and 10 mm.

**Figure 9.**
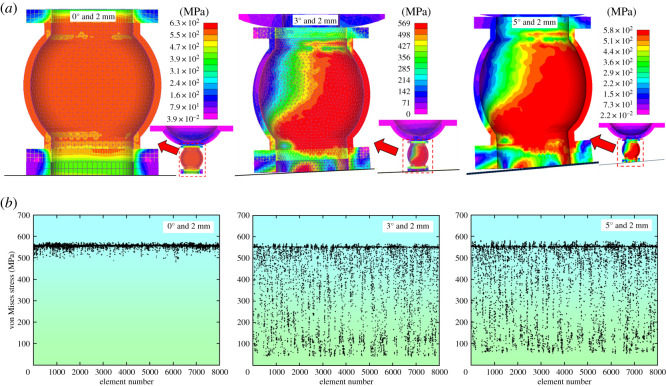
von Mises stress at the displacement of 2 mm. (*a*) The von Mises stress distribution characteristics. (*b*) Element's von Mises stress.

Figure 9*a* shows the von Mises stress distribution laws under various eccentric loading conditions when the deformation is 2 mm. To analyse the distribution characteristics quantitatively, the von Mises stresses of yielding pipe's 7995 tetrahedral elements were given, as shown in figure 9*b*.

Figure 9*a* indicates that when the eccentric load is 0°, von Mises stresses distribute evenly within the yielding pipe. When the eccentric loads are 3° and 5° off the centre line, von Mises stresses concentrate at the right side of the yielding pipe. Therefore, eccentric load could affect the yielding pipe's utilization rate at the initial stage of deformation. Figure 9*b* shows that when the eccentric load coincides with the centre line (0° condition), von Mises stresses concentrate between 500 and 550 MPa. However, when the eccentric loads are 3° and 5°, the von Mises stresses fluctuate between 50 and 550 MPa, implying that eccentric load have impacts on the utilization rate of the yielding pipe at the initial stage of deformation.

**Figure 10.**
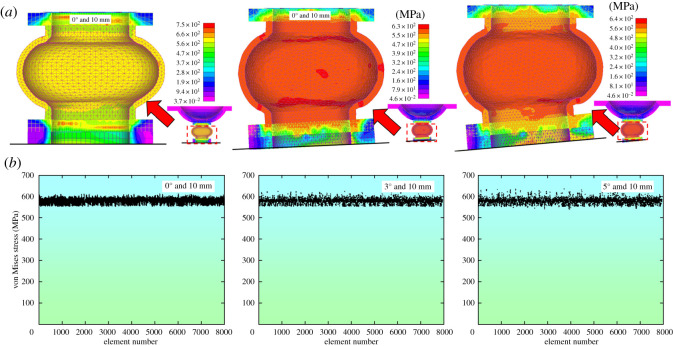
The von Mises stresses with the deformation of 10 mm. (*a*) The von Mises stress distribution characteristics. (*b*) Element's von Mises stress.

Similarly, figure 10*a* shows von Mises stress distribution under different eccentric load when the displacement is 10 mm. Figure 10*b* gives the von Mises stress values of 7995 tetrahedral elements within the yielding pipe.

In figure 10*a*, it could be seen that the von Mises stresses distribute evenly under various eccentric load, indicating that the utilization rate of the yielding pipe is higher at the later stage of deformation. In figure 10*b*, the von Mises stresses concentrate between 500 and 550 MPa, suggesting that eccentric load having fewer impacts on the utilization rate of the yielding pipe.

**Figure 11.**
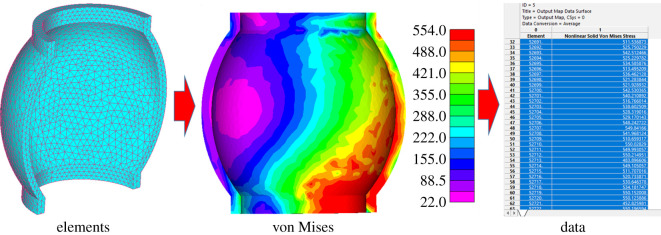
The way of extracting elements' von Mises stresses.

To further analyse the utilization rate, the elements' von Mises stresses were constantly extracted, and the data were exported after the meshing of the finite model, as shown in figure 11.

**Figure 12.**
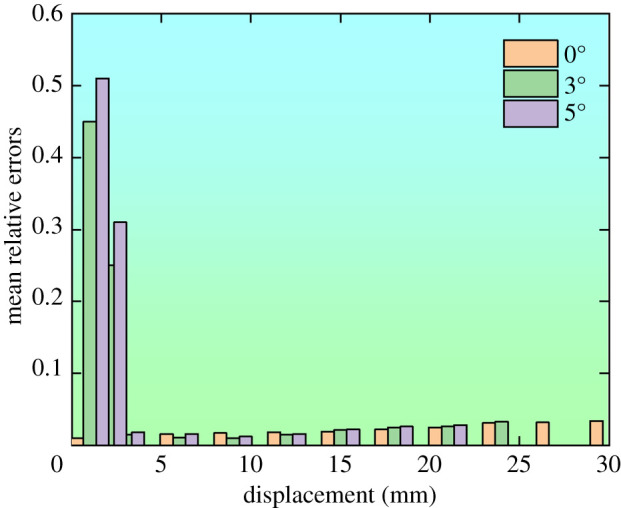
The von Mises stresses of the yielding pipe.

A C++ program was adopted to complete the process of extracting the von Mises stress data. Von Mises stresses of 7995 tetrahedron elements were recorded and figure 12 displays their relative average deviations under different displacement.

Figure 12 indicates that:

(1) When eccentric loads are 3° and 5°, within the displacement range of 0–2 mm, the mean relative errors of the von Mises stresses are relatively large, the stresses are uneven in the pipe and the utilization rate is low. When the displacement falls between 5 and 24 mm, the mean relative errors are within 4%. The stresses are uniformly distributed, and the utilization rate is high.
(2) When the eccentric load is 0°, the mean relative errors of von Mises stresses are within 4%. This indicates the stresses are uniform and the yielding pipe with current material and structural design can function effectively in terms of the utilization rate.

### 4.3. Plastic properties of bolt's components

**Figure 13.**
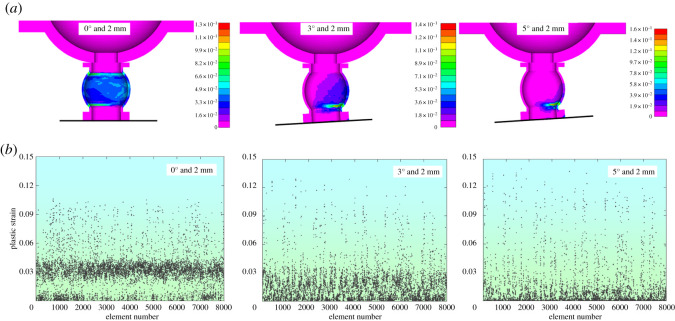
Plastic strains at the displacement of 2 mm. (*a*) Distribution characteristics of the plastic strain. (*b*) The element's plastic strains.

During the yielding process of the pre-stressed yielding bolt, plastic failure of the yielding pipe is common. Meanwhile, other components should be at the elastic stage. This section analyses how eccentric load influences plastic properties of the yielding bolt's components. The plastic strains of the yielding pipe were extracted at the displacement of 2 mm and are displayed in figure 13.

Figure 13 shows that the plastic strains of the bolt's components concentrate in the yielding pipe under different eccentric loads, while other components are basically at the elastic stage. When the eccentric load is 0°, the plastic zone in the yielding pipe is evenly distributed. When eccentric loads are 3° and 5°, the plastic zone concentrates on the right side of the pipe at the initial stage of displacement.

**Figure 14.**
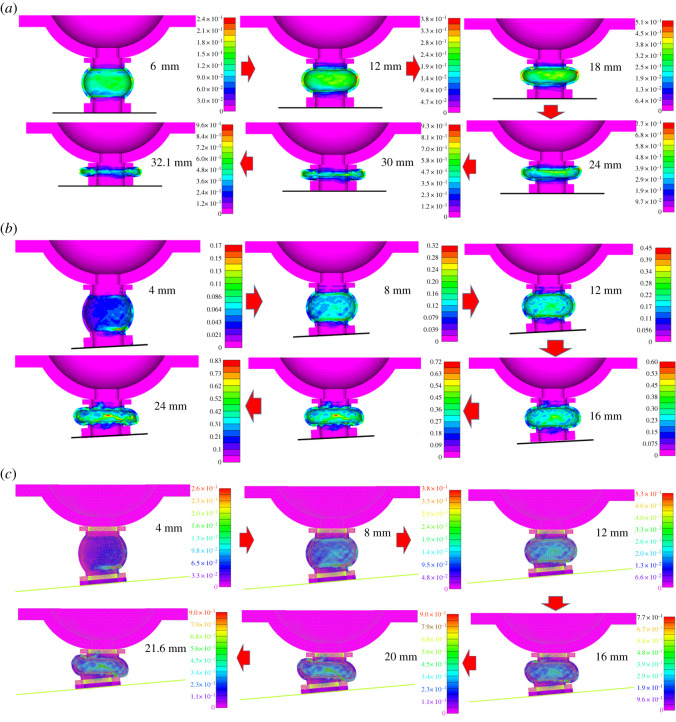
Plastic strains of the bolt components. (*a*) 0°. (*b*) 3°. (*c*) 5°.

The plastic strains with different displacement were extracted for further analysis, as shown in figure 14.

From figure 14, it could be summarized that von Mises stresses of the bolt components concentrate in the yielding pipe with various displacements, while other components are basically at the elastic stage. The plastic strain distribution characteristics are significantly affected by eccentric load at the initial stage of displacement, while at the later stage, eccentric loads mainly concentrate in the middle of the yielding pipe.

Above all, eccentric loads influence the bolt components' plastic strain distribution characteristics at the initial stage of displacement, while they have a negligible effect at later stage.

### 4.4. Absorptive capacity of the yielding pipe

**Figure 15.**
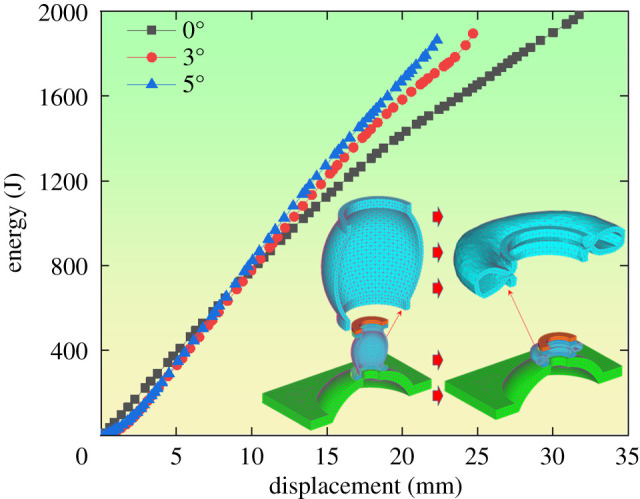
Law of the absorbed energy by the yielding pipe with various eccentric loads.

In the roadway suffering from high stress and large deformation, surrounding rocks release energy during its deformation and the yielding pipe absorbs part of the deformation energy. The amount and rate of energy absorbed could reflect the performance of the pre-stressed yielding bolt. As shown in figure 15, C++ programming adds the absorbed elastic energy and plastic energy within the element together and obtains the overall amount of energy absorbed by the yielding pipe.

Figure 15 indicates that:

(1) During the yielding process, when eccentric loads are 0°, 3° and 5°, the energy absorbed by half of the yielding pipe is 2021, 1894 and 1863 J, respectively. Thus, the energy absorbed by the whole yielding pipe is doubled. As the eccentric load increases, the absorbed energy will reduce, basically no more than 10%.
(2) At the initial stage of the displacement, when the eccentric load is 0°, the energy absorbed by the yielding pipe is larger than that when eccentric loads are 3° and 5°. On the contrary, it is the opposite at the later stage.
(3) The rate of energy absorbed remains basically stable under various eccentric loading conditions, which proves that the structure of the yielding pipe is stable during the yielding process.

## 5. Discussion

Based on the analysis in §4, eccentric load could affect the utilization rate of the yielding bolts during the initial deformation, while these impacts can be neglected during later deformation. At this stage, eccentric loads mainly affect the yielding bolt's displacement–load relations. That is mainly reflected by the value of yielding point, its corresponding displacement and the yielding amount. The following case could provide further explanation.

Large deformation of the roadway occurred during the mining of the working face in Tiefa Mining Area, located in Diaobingshan city, Liaoning province, China. The yielding bolts were used to tackle the problem. The yielding point was larger than 150 kN and the yielding amount was larger than 25 mm. According to table 2, the yielding bolt in MSGLW-500/20(RY) series was selected with the yielding point of 160 kN and the yielding amount of 25 mm, which could have met the design requirements. However, the on-site borehole deviation was between 3° and 5°. According to the analysis in §4, when the borehole deviation is between 3 and 5°, the yielding point should be between 180.2 and 194.0 kN. The yielding amount should be between 21.0 and 23.5 mm. Thus, the yielding amount in this case could not satisfy the design requirement due to the borehole deviation.

In the field, the borehole deviation was adjusted to control the yielding amount and point. According to influence laws of eccentric loads on the yielding bolt's displacement–load relations, the following relationship exists between the yielding amount and eccentric loads: *L*_2_ = −2.0229*θ* + 30.224. Therefore, the eccentric load was controlled between 0 and 2.5°. At this point, the MSGLW-500/20(RY) yielding bolt's yielding point was between 160.75 and 177.76 kN, and the yielding amount was between 25.2 and 30.2 mm, which could meet the design requirement.

## 6. Conclusion

In this paper, the yielding bolt's numerical analysis model was first established with the numerical simulation method. Then, the impacts of eccentric load on displacement–load relations, utilization rate of the yielding pipe, plastic strains of bolt components and the absorptive capacity of the yielding pipe were analysed in detail. Three conclusions are drawn as below:

(1) Eccentric loads affected the utilization rate of the yielding pipe, plastic strains of bolt components and the absorptive capacity to a certain degree at the initial stage of displacement, while these impacts could be neglected in later deformation.
(2) Eccentric loads mainly affected the displacement–load relations of the yielding bolt. As the eccentric load increased, the yielding point and its corresponding displacement increased linearly, while the yielding amount decreased linearly.
(3) In the field, eccentric loads could be adjusted to control the yielding point and amount of the yielding bolt in order to meet the design requirements of roadway support.

## Supplementary Material

Reviewer comments

## Data accessibility

Data available from the Dryad Digital Repository: https://doi.org/10.5061/dryad.34tmpg4gg [33].
