# Supplementary material for: The influence law of eccentric load on the performance of yielding bolt
Source: R Soc Open Sci. 2020 Jul 1;7(7):200227. doi: 10.1098/rsos.200227 (PMC7428256; doi:10.1098/rsos.200227)
Supplement: Reviewer comments [file rsos200227_review_history.pdf]

THE ROYAL SOCIETY
PUBLISHING

ROYAL SOCIETY
OPEN SCIENCE

The influence law of eccentric load on the performance of yielding bolt

Yang Tai, Hongchun Xia, Shaoping Huang, Jie Meng and Wei Li

Article citation details
R. Soc. open sci. **7**: 200227.
http://dx.doi.org/10.1098/rsos.200227

Review timeline

Original submission:	12 February 2020
1st revised submission:	27 May 2020
2nd revised submission:	13 June 2020
Final acceptance:	16 June 2020

Note: Reports are unedited and appear as submitted by the referee. The review history appears in chronological order.

Review History

RSOS-200227.R0 (Original submission)

Review form: Reviewer 1

Is the manuscript scientifically sound in its present form?
Yes

Are the interpretations and conclusions justified by the results?
Yes

Is the language acceptable?
Yes

Do you have any ethical concerns with this paper?
No

Have you any concerns about statistical analyses in this paper?
No

Recommendation?
Accept with minor revision (please list in comments)

Reports © 2020 The Reviewers; Decision Letters © 2020 The Reviewers and Editors; Responses © 2020 The Reviewers, Editors and Authors. Published by the Royal Society under the terms of the Creative Commons Attribution License http://creativecommons.org/licenses/by/4.0/, which permits unrestricted use, provided the original author and source are credited

Comments to the Author(s)
The paper is well written, and addressing an important technical issue in mining industry. There are some minor grammatical issues that could be easily fixed. Please see my comments in annotated copy (Appendix A).
However, the paper requires a major literature review, which hasn't been done properly.

Review form: Reviewer 2

Is the manuscript scientifically sound in its present form?
No

Are the interpretations and conclusions justified by the results?
No

Is the language acceptable?
No

Do you have any ethical concerns with this paper?
No

Have you any concerns about statistical analyses in this paper?
No

Recommendation?
Major revision is needed (please make suggestions in comments)

Comments to the Author(s)
Thank you for submitting your paper.
While the intent of the paper in furthering understanding of the behaviour of (rock)bolts that will contribute to an improvement in the stability of underground roadways and hence enhancing safety in mines is commendable, the paper needs extensive editing (see Appendix B).

Decision letter (RSOS-200227.R0)

30-Apr-2020

Dear Dr Tai,

The editors assigned to your paper ("The Influence Law of Eccentric Load on the Performance of Yielding Bolt") have now received comments from reviewers. We would like you to revise your paper in accordance with the referee and Associate Editor suggestions which can be found below (not including confidential reports to the Editor). Please note this decision does not guarantee eventual acceptance.

Please submit a copy of your revised paper before 23-May-2020. Please note that the revision deadline will expire at 00.00am on this date. If we do not hear from you within this time then it will be assumed that the paper has been withdrawn. In exceptional circumstances, extensions may be possible if agreed with the Editorial Office in advance. We do not allow multiple rounds of revision so we urge you to make every effort to fully address all of the comments at this stage.

If deemed necessary by the Editors, your manuscript will be sent back to one or more of the original reviewers for assessment. If the original reviewers are not available, we may invite new reviewers.

To revise your manuscript, log into http://mc.manuscriptcentral.com/rsos and enter your Author Centre, where you will find your manuscript title listed under "Manuscripts with Decisions." Under "Actions," click on "Create a Revision." Your manuscript number has been appended to denote a revision. Revise your manuscript and upload a new version through your Author Centre.

When submitting your revised manuscript, you must respond to the comments made by the referees and upload a file "Response to Referees" in "Section 6 - File Upload". Please use this to document how you have responded to the comments, and the adjustments you have made. In order to expedite the processing of the revised manuscript, please be as specific as possible in your response.

In addition to addressing all of the reviewers' and editor's comments please also ensure that your revised manuscript contains the following sections as appropriate before the reference list:

• Ethics statement (if applicable)
If your study uses humans or animals please include details of the ethical approval received, including the name of the committee that granted approval. For human studies please also detail whether informed consent was obtained. For field studies on animals please include details of all permissions, licences and/or approvals granted to carry out the fieldwork.

• Data accessibility
It is a condition of publication that all supporting data are made available either as supplementary information or preferably in a suitable permanent repository. The data accessibility section should state where the article's supporting data can be accessed. This section should also include details, where possible of where to access other relevant research materials such as statistical tools, protocols, software etc can be accessed. If the data have been deposited in an external repository this section should list the database, accession number and link to the DOI for all data from the article that have been made publicly available. Data sets that have been deposited in an external repository and have a DOI should also be appropriately cited in the manuscript and included in the reference list.

If you wish to submit your supporting data or code to Dryad (http://datadryad.org/), or modify your current submission to dryad, please use the following link:
http://datadryad.org/submit?journalID=RSOS&manu=RSOS-200227

• Competing interests
Please declare any financial or non-financial competing interests, or state that you have no competing interests.

• Authors’ contributions
All submissions, other than those with a single author, must include an Authors’ Contributions section which individually lists the specific contribution of each author. The list of Authors should meet all of the following criteria; 1) substantial contributions to conception and design, or acquisition of data, or analysis and interpretation of data; 2) drafting the article or revising it critically for important intellectual content; and 3) final approval of the version to be published.

All contributors who do not meet all of these criteria should be included in the acknowledgements.

We suggest the following format:

AB carried out the molecular lab work, participated in data analysis, carried out sequence alignments, participated in the design of the study and drafted the manuscript; CD carried out the statistical analyses; EF collected field data; GH conceived of the study, designed the study, coordinated the study and helped draft the manuscript. All authors gave final approval for publication.

• Acknowledgements
Please acknowledge anyone who contributed to the study but did not meet the authorship criteria.

• Funding statement
Please list the source of funding for each author.

Once again, thank you for submitting your manuscript to Royal Society Open Science and I look forward to receiving your revision. If you have any questions at all, please do not hesitate to get in touch.

Kind regards,
Anita Kristiansen
Editorial Coordinator

Royal Society Open Science
openscience@royalsociety.org

on behalf of Professor Zach Agioutantis (Associate Editor) and R. Kerry Rowe (Subject Editor)
openscience@royalsociety.org

Comments to Author:

Reviewers' Comments to Author:
Reviewer: 1

Comments to the Author(s)
The paper is well written, and addressing an important technical issue in mining industry. There are some minor grammatical issues that could be easily fixed. Please see my comments in annotated copy (attached (RSOS-200227_Proof_hi.pdf)).
However, the paper requires a major literature review, which hasn't been done properly.

Reviewer: 2

Comments to the Author(s)
Thank you for submitting your paper.
While the intent of the paper in furthering understanding of the behaviour of (rock)bolts that will contribute to an improvement in the stability of underground roadways and hence enhancing safety in mines is commendable, the paper needs extensive editing. (rsos-200227_proof_hi_comments.pdf) (rsos-200227_proof_hi_summary.pdf)

Author's Response to Decision Letter for (RSOS-200227.R0)

See Appendix C.

Decision letter (RSOS-200227.R1)

05-Jun-2020

Dear Dr Tai:

On behalf of the Editors, I am pleased to inform you that your Manuscript RSOS-200227.R1 entitled "The Influence Law of Eccentric Load on the Performance of Yielding Bolt" has been accepted for publication in Royal Society Open Science subject to minor revision in accordance with the referee suggestions. Please find the referees' comments at the end of this email.

The reviewers and Subject Editor have recommended publication, but also suggest some minor revisions to your manuscript. Therefore, I invite you to respond to the comments and revise your manuscript.

• Ethics statement
If your study uses humans or animals please include details of the ethical approval received, including the name of the committee that granted approval. For human studies please also detail whether informed consent was obtained. For field studies on animals please include details of all permissions, licences and/or approvals granted to carry out the fieldwork.

• Data accessibility
It is a condition of publication that all supporting data are made available either as supplementary information or preferably in a suitable permanent repository. The data accessibility section should state where the article's supporting data can be accessed. This section should also include details, where possible of where to access other relevant research materials such as statistical tools, protocols, software etc can be accessed. If the data has been deposited in an external repository this section should list the database, accession number and link to the DOI for all data from the article that has been made publicly available. Data sets that have been deposited in an external repository and have a DOI should also be appropriately cited in the manuscript and included in the reference list.

If you wish to submit your supporting data or code to Dryad (http://datadryad.org/), or modify your current submission to dryad, please use the following link:
http://datadryad.org/submit?journalID=RSOS&manu=RSOS-200227.R1

• Competing interests
Please declare any financial or non-financial competing interests, or state that you have no competing interests.

• Authors' contributions
All submissions, other than those with a single author, must include an Authors' Contributions section which individually lists the specific contribution of each author. The list of Authors should meet all of the following criteria; 1) substantial contributions to conception and design, or acquisition of data, or analysis and interpretation of data; 2) drafting the article or revising it critically for important intellectual content; and 3) final approval of the version to be published.

All contributors who do not meet all of these criteria should be included in the acknowledgements.

We suggest the following format:
AB carried out the molecular lab work, participated in data analysis, carried out sequence alignments, participated in the design of the study and drafted the manuscript; CD carried out the statistical analyses; EF collected field data; GH conceived of the study, designed the study,

coordinated the study and helped draft the manuscript. All authors gave final approval for publication.

• Acknowledgements
Please acknowledge anyone who contributed to the study but did not meet the authorship criteria.

• Funding statement
Please list the source of funding for each author.

Please note that we cannot publish your manuscript without these end statements included. We have included a screenshot example of the end statements for reference. If you feel that a given heading is not relevant to your paper, please nevertheless include the heading and explicitly state that it is not relevant to your work.

Because the schedule for publication is very tight, it is a condition of publication that you submit the revised version of your manuscript before 14-Jun-2020. Please note that the revision deadline will expire at 00.00am on this date. If you do not think you will be able to meet this date please let me know immediately.

To revise your manuscript, log into https://mc.manuscriptcentral.com/rsos and enter your Author Centre, where you will find your manuscript title listed under "Manuscripts with Decisions". Under "Actions," click on "Create a Revision." You will be unable to make your revisions on the originally submitted version of the manuscript. Instead, revise your manuscript and upload a new version through your Author Centre.

When submitting your revised manuscript, you will be able to respond to the comments made by the referees and upload a file "Response to Referees" in "Section 6 - File Upload". You can use this to document any changes you make to the original manuscript. In order to expedite the processing of the revised manuscript, please be as specific as possible in your response to the referees.

When uploading your revised files please make sure that you have:

1) A text file of the manuscript (tex, txt, rtf, docx or doc), references, tables (including captions) and figure captions. Do not upload a PDF as your "Main Document".
2) A separate electronic file of each figure (EPS or print-quality PDF preferred (either format should be produced directly from original creation package), or original software format)
3) Included a 100 word media summary of your paper when requested at submission. Please ensure you have entered correct contact details (email, institution and telephone) in your user account
4) Included the raw data to support the claims made in your paper. You can either include your data as electronic supplementary material or upload to a repository and include the relevant doi within your manuscript
5) All supplementary materials accompanying an accepted article will be treated as in their final form. Note that the Royal Society will neither edit nor typeset supplementary material and it will be hosted as provided. Please ensure that the supplementary material includes the paper details where possible (authors, article title, journal name).

Supplementary files will be published alongside the paper on the journal website and posted on the online figshare repository (https://figshare.com). The heading and legend provided for each supplementary file during the submission process will be used to create the figshare page, so please ensure these are accurate and informative so that your files can be found in searches. Files on figshare will be made available approximately one week before the accompanying article so that the supplementary material can be attributed a unique DOI.

Once again, thank you for submitting your manuscript to Royal Society Open Science and I look forward to receiving your revision. If you have any questions at all, please do not hesitate to get in touch.

Kind regards,
Andrew Dunn
Royal Society Open Science Editorial Office
Royal Society Open Science
openscience@royalsociety.org

on behalf of Professor Zach Agioutantis (Associate Editor) and R. Kerry Rowe (Subject Editor)
openscience@royalsociety.org

Associate Editor Comments to Author (Professor Zach Agioutantis):

The authors have responded to the reviewers comments and recommendations. However, although the text seems to have been reviewed for English, there are still a lot odd expressions in the text. The authors need to provide proof of editorial review

In addition, the authors need to make the following changes
a) change "Mises stress" to "von Mises stress"
b) units in tables and graphs should be provided in (), i.e. (MPa) and not /MPa. Please consult the English literature on how units are represented
c) The inner and outer diameter of the yielding pipe are not given. Table 2 provides 20 and 22 mm for the yielding bolt, and provides the yielding pipe height but not the inner and outer diameters.
d) What does "Materials" in Table 2 correspond to? Is that the yield strength of the steel? If so, adjust the heading in the table accordingly.
e) bolt diameter is a better expression compared to bolt's diameter (both are in the text)
f) tray in figure 6 should be plate. Consult the international literature
g) "All main elements surface" is not a good expression, please rephrase

Author's Response to Decision Letter for (RSOS-200227.R1)

See Appendix D.

Decision letter (RSOS-200227.R2)

16-Jun-2020

Dear Dr Tai,

It is a pleasure to accept your manuscript entitled "The Influence Law of Eccentric Load on the Performance of Yielding Bolt" in its current form for publication in Royal Society Open Science.

Please ensure that you send to the editorial office an editable version of your accepted manuscript, and individual files for each figure and table included in your manuscript. You can send these in a zip folder if more convenient. Failure to provide these files may delay the

processing of your proof. You may disregard this request if you have already provided these files to the editorial office.

You can expect to receive a proof of your article in the near future. Please contact the editorial office (openscience_proofs@royalsociety.org) and the production office (openscience@royalsociety.org) to let us know if you are likely to be away from e-mail contact -- if you are going to be away, please nominate a co-author (if available) to manage the proofing process, and ensure they are copied into your email to the journal.

Due to rapid publication and an extremely tight schedule, if comments are not received, your paper may experience a delay in publication. Royal Society Open Science operates under a continuous publication model. Your article will be published straight into the next open issue and this will be the final version of the paper. As such, it can be cited immediately by other researchers. As the issue version of your paper will be the only version to be published I would advise you to check your proofs thoroughly as changes cannot be made once the paper is published.

Please see the Royal Society Publishing guidance on how you may share your accepted author manuscript at https://royalsociety.org/journals/ethics-policies/media-embargo/.

Thank you for your fine contribution. On behalf of the Editors of Royal Society Open Science, we look forward to your continued contributions to the Journal.

Kind regards,
Royal Society Open Science Editorial Office
Royal Society Open Science
openscience@royalsociety.org

on behalf of Professor Zach Agioutantis (Associate Editor) and R. Kerry Rowe (Subject Editor)
openscience@royalsociety.org

Follow Royal Society Publishing on Twitter: @RSocPublishing
Follow Royal Society Publishing on Facebook:
https://www.facebook.com/RoyalSocietyPublishing.FanPage/
Read Royal Society Publishing's blog: https://blogs.royalsociety.org/publishing/

Appendix A

ROYAL SOCIETY
OPEN SCIENCE

The Influence Law of Eccentric Load on the Performance of Yielding Bolt

Journal:	*Royal Society Open Science*
Manuscript ID	RSOS-200227
Article Type:	Research
Date Submitted by the Author:	12-Feb-2020
Complete List of Authors:	Tai, Yang; China University of Mining and Technology,<br>Xia, Hongchun; Dalian University<br>Huang, Shaopin; China University of Geosciences<br>Meng, Jie; University of Ottawa<br>Li, Wei; Dalian University
Subject:	Energy < ENGINEERING AND TECHNOLOGY, Engineering geology < ENGINEERING AND TECHNOLOGY, Mechanical engineering < ENGINEERING AND TECHNOLOGY
Keywords:	Yielding bolt, eccentric load, performance, numerical simulation
Subject Category:	Engineering

SCHOLARONE™
Manuscripts

Author-supplied statements

Relevant information will appear here if provided.

Ethics

Does your article include research that required ethical approval or permits?:
This article does not present research with ethical considerations

Statement (if applicable):
CUST_IF_YES_ETHICS :No data available.

Data

It is a condition of publication that data, code and materials supporting your paper are made publicly available. Does your paper present new data?:
Yes

Statement (if applicable):
Data available from the Dryad Digital Repository: https:// doi:10.5061/dryad.34tmpg4gg [25]. Dryad review URL: https://datadryad.org/stash/share/TD5tUaCfaCiJYAlxXq1Yi34CpcOuhIaE9fXXH01M9Zo.

Conflict of interest

I/We declare we have no competing interests

Statement (if applicable):
CUST_STATE_CONFLICT :No data available.

Authors' contributions

This paper has multiple authors and our individual contributions were as below

Statement (if applicable):
Y.T. participated in the design of the study and drafted the manuscript; H.X. carried out the statistical analyses; S.H. collected field data; J.M. conceived of the study and designed the study; W.L. coordinated the study and helped draft the manuscript. All authors gave final approval for publication.

The Influence Law of Eccentric Load on the Performance of Yielding Bolt

Yang Tai [1], Hongchun Xia [2,*], Shaoping Huang [3,*], Jie Meng [4], Wei Li [2]

1. School of Mines, China University of Mining and Technology, Xuzhou 221116, China;
2. College of Civil Engineering and Architecture, Dalian University, Liaoning, Dalian 116622, China;
3. Faculty of Engineering, China University of Geosciences, Wuhan, Hubei 430074, China;
4. Department of Civil Engineering, University of Ottawa, 161 Colonel by, Ottawa (Ontario) K1N 6N5, Canada.

* Correspondence author: cumtcqty@gmail.com

Abstract: In order to adapt high stress and large deformation of roadways, the prestressed yielding bolt has been developed. However, borehole deviation makes the boreholes out of perpendicular to the roadway surface, and the prestressed yielding bolt will be under eccentric load. In order to reveal the influence laws of eccentric load on the performance of the prestressed yielding bolt, the numerical simulation was carried out in this paper. Then, the influence of eccentric load on displacement-load relations, use efficiency for the yielding pipe material, plastic strains of the bolt components, as well as the absorptive capacity of the yielding pipe were analyzed. The results are as follows: (1). Eccentric loads affected use efficiency of the yielding pipe material, plastic strains of bolt components and the absorptive capacity to a certain degree at the stage of initial displacement, while these impacts could be neglected at the stage of the later displacement; (2) Eccentric load mainly affected the yielding pipe's displacement-load relations. As the eccentric load increased, the yielding point and its corresponding displacement increased linearly while the yielding amount decreased linearly; (3) The eccentric load could be adjusted to control the yielding point and amount in order to meet the roadway support's requirement for the yielding bolt.

Keywords: Yielding bolt; eccentric load; performance; numerical simulation

1 Introduction

With the advantages of sound control effects, low costs and simple operation, the bolt is one of the most common support methods for surrounding rocks in coal mines [1, 2]. As the mining depth of coal mines increases, the roadway surrounding rocks are characterized with high stress and large deformation [3]. At this time, common bolts are difficult to meet the support requirements, so the structures of bolts have been optimized. Moreover, various types of extensible bolts have also been design. Among them, the most common type is the yielding bolt developed by American Jennmar Corporation, as shown in Figure 1 (a). It could be seen that the yielding bolt consists of nuts, the yielding pipe, tray and high-intensity rod body. As one of the core components, the yielding pipe is made of seamless steel pipe. The excessive deformation energy could be released through the deformation of the yielding pipe. After the yielding, the yielding bolt has stronger constraint forces and could effectively control the roadway surrounding rock deformation and ensure the stability of the roadway [4].

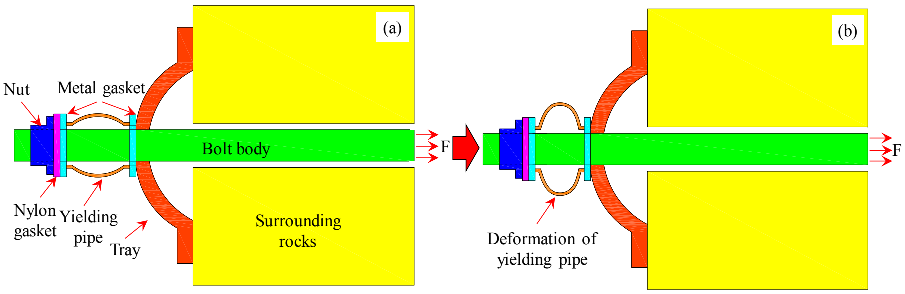

Fig.1 The structure of the yielding bolt

Before installation of the yielding bolt, professional drilling equipment should be used to drill holes with certain depths and diameters. Due to equipment accuracy and personal operations, it is inevitable to occur hole deviations

during the drilling process, as shown in Figure 2. In this case, the yielding bolt will occur eccentric load.

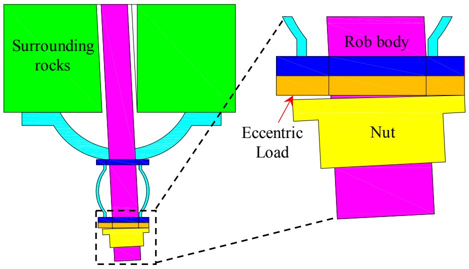

Fig.2 The eccentric load for yielding bolt

In-depth studies have been made on the yielding bolt by scholars both at home and abroad, mainly focusing on evaluation of the yielding effects, selection of key parameters of the yielding bolt, and coupling relationships between the yielding bolt and the surrounding rock. **(1) The evaluation of the yielding effects.** Xiang et al. [5] established a mechanical model of the yielding bolt. The finite element shear strength reduction method was used to analyze and evaluate the support effects of the high-intensity yielding bolt; Zhu et al. [6] built a constitutive model of the yielding anchor. Then, FLAC^{3D} software was used to analyze the slope reinforcement effects of the anchor; Yang [7] introduced the structure and the support process of the large-scale yielding bolt, and analyzed its performance characteristics at the stages of elastic deformation, sliding and yielding by ANSYS software. Meanwhile, the yielding results were also evaluated; Lian et al. [8] built a 3D mechanical model with the consideration of the shear failure between the prestressed yielding bolt and the rock, and developed the corresponding calculation method. **(2) The selection key parameters of the yielding bolt.** Shan et al. [9] divided the tensile process of the yielding bolt into the elastic stage, the yielding stage and the plastic hardening stage. Based on the energy constitutive equations at different stages, the ultimate load calculation formula was given; Yang [10] discussed various yielding bolts' basic mechanical parameters such as the yielding point, yielding load stability, the maximum yielding amount etc., which could provide basis for the selection of the yielding bolt. **(3) The coupling relationships between the yielding bolt and the surrounding rock.** Guo et al. [11] introduced a new type of pressure-dispersive yielding bolt. Its mechanism and mechanical behaviors were analyzed, and the coupling relationships between the yielding bolt and the surrounding rock were given; Zhang et al. [12] systematically analyzed variations of surrounding rock stresses, displacements and axial forces before and after the support of the yielding bolt.

The studies mainly focus on evaluation of the yielding effects, the key parameters of the yielding bolt as well as coupling relationships between the yielding bolt and the surrounding rock. However, the influence laws of eccentric load on the performance of the yielding bolt have not been studied. In this paper, the numerical model of the yielding bolt was established. Then the influence laws of eccentric loads on the performance of the yielding bolt were explored. At the same time, the engineering design was carried out.

2 Numerical calculation method

2.1 Calculation method

2.1.1 Method of solving nonlinear equations

During the yielding process of the yielding bolt, multiple nonlinearities are involved, such as the geometric nonlinearity, material nonlinearity, contact nonlinearity etc. In addition, the bolt's structure is complex. Therefore, the traditional theoretical calculations could not reveal influence laws of eccentric load on the performance of the yielding bolt. Thus, the numerical simulation was adopted, whose basic flow is shown in Figure 3.

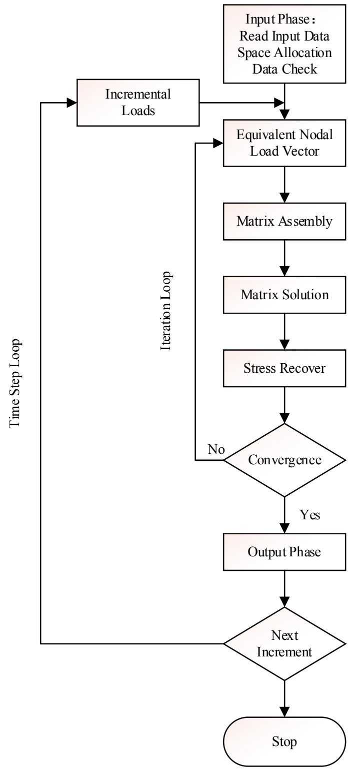

Fig.3 Diagram of solutions

It could be seen that the steps of the matrix solution involve many nonlinear equations, so how to solve the nonlinear equations is one of the core steps for the numerical calculation.

A nonlinear system is expressed as:

$$K^T \Delta u = F - R = r \tag{1}$$

Where, K is the elastic stiffness matrix, K^T is the tangent stiffness matrix, and Δu is the displacement vector. F is the applied load vector, R is the internal nodal-load vector, and r is the residual.

One method to solve nonlinear problems is to use iterations. The objective of the iterative techniques is to minimize the function. In the simplest method,

$$u_{k+1} = u_k + \alpha_k r_k \tag{2}$$

Where, u_k and u_{k+1} are the displacement vectors at the kth iteration and (k+1) th iteration, and r_k is the residual at the kth iteration.

$$\alpha_k = r_k^T r_k / r_k^T K r_k \tag{3}$$

The problem is that the gradient directions are too close, thus resulting in poor convergence.

An improved method led to the *conjugate gradient method*, in which

$$u_{k+1} = u_k + \alpha_k p_k \tag{4}$$

$$\alpha_k = p_k^T r_{k-1} / p_k^T K r_k \tag{5}$$

The trick is to choose p_k to be K conjugate to p_1, p_2, ... p_{k-1}. Hence, named *conjugate gradient methods*. Note the elegance of these methods is that the solution may be obtained through a series of matrix multiplications and the stiffness matrix never needs to be decomposed.

2.1.2 The iterative method

The Newton-Raphson method was used in the numerical simulation. Its basis requirement in structural analysis

is that equilibrium be satisfied. Considering the following set of equations:

$$K^T(u)\delta u = F - R(u) \tag{6}$$

Where, u is the nodal displacement vector, F is the applied load vector, R is the internal nodal-load vector, and K^T is the tangent stiffness matrix. The applied load vector is obtained from internal stresses as:

$$R = \sum_{\text{elem}} \int_V \beta^T \sigma \mathrm{d}v \tag{7}$$

In this set of equations, both R and K^T are functions of u. In many cases, F is also a function of u (for example, if F follows pressure loads, the nodal load vector is a function of the orientation of the structure). The equations suggest that use of the full Newton-Raphson method is appropriate.

Suppose that the last obtained approximate solution is termed δu^i, where i indicates the iteration number. Equation (6) can then be written as:

$$K^T\left(u_{\text{n+1}}^{i-1}\right)\delta u^i = F - R\left(u_{\text{n+1}}^{i-1}\right) \tag{8}$$

This equation is solved for δu^i and the next appropriate solution is obtained by

$$\Delta u^i = \Delta u^{i-1} + \delta u^i \quad \text{and} \quad u_{\text{n+1}}^i = u_{n+1}^{i-1} + \delta u^i \tag{9}$$

Solution of this equation completes one iteration, and the process can be repeated. The subscript n denotes the increment number. Unless stated otherwise, the subscript n+1 is dropped with all quantities referring to the current state.

2.2 Contact method

Multiple contacts involve in the numerical simulation, such as contacts among the nylon gasket, metal gasket, the yielding pipe and the tray. In addition, the yielding pipe may occur self-contact during the yielding process. According to Newton's third law, the contact surface should satisfy:

$$\begin{cases} t_N^A + t_N^B = 0 \\ t_T^A + t_T^B = 0 \end{cases} \tag{10}$$

where t_N^A and t_N^B are object normal contact forces of A and B, respectively; t_T^A and t_T^B are object tangential contact forces of A and B, respectively. The contact was realized by the symmetric penalty function method of finite elements, and the main calculation steps are as follows:

I. As shown in Figure 4, to search the closest host node m_s for any slave node n_s.

II. To check all main elements' surfaces related with the master node m_s and determine the possible contact main elements' surfaces when the slave node n_s penetrates the main elements. If the master node m_s does not coincide with the slave node, and formula (11) is satisfied, the node n_s will contact with the main element surface S_i.

$$\begin{cases} \left(C_{\text{i}} \times S_i\right)\cdot\left(C_{\text{i}} \times C_{\text{i+1}}\right) > 0 \\ \left(C_{\text{i}} \times S_i\right)\cdot\left(S_i \times C_{\text{i+1}}\right) > 0 \end{cases} \tag{11}$$

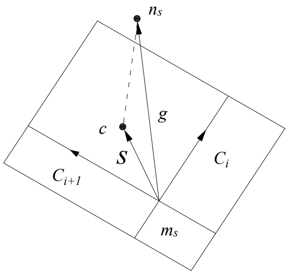

Fig.4 All main elements' surfaces related with m_s

In formula (11), C_i and C_{i+1} are two edge vectors at the node m_s in the main element surface. Vector **S** is the projection of vector ***g*** on the main element surface, and ***g*** is the vector from the master node m_s to the slave node n_s.

$$S = g - (gm)m\ ,\quad m = C_i C_{i+1} \big/ \left| C_i\ C_{i+1} \right| \tag{12}$$

If node n_s is in or near the intersection between two elements' surfaces, the above inequation may be uncertain and the following formula could be used:

$$S = \max\left(gC_i / \left| C_i \right| \right) \quad i = 1, 2, ... \tag{13}$$

III. To determine the position of the contact point c of the slave node n_s in the main element's surface. The position vector ***p*** at any point in the main element surface could be expressed as follows:

$$p = f_1(\xi,\eta)i_1 + f_2(\xi,\eta)i_2 + f_3(\xi,\eta)i_3 \tag{14}$$

Where, $f_i(\xi,\eta) = \sum_{j=1}^{4} \phi_j(\xi,\eta) x_i^j\ , \phi_j(\xi,\eta) = (1+\xi_j\xi)(1+\eta_j\eta)/4$ and x_i^j is the x_i coordinate value of j th node of the element; i_1, i_2, i_3 are position vectors of x_1, x_2, x_3 with the coordinates. The position of the contact point $c(\xi_c\ ,\eta_c)$ is the solution of the following formulas.

$$\begin{cases} \dfrac{\partial p}{\partial \xi}(\xi_c,\eta_c)\cdot\left[t - p(\xi_c,\eta_c) \right] = 0 \\ \dfrac{\partial p}{\partial \eta}(\xi_c,\eta_c)\cdot\left[t - p(\xi_c,\eta_c) \right] = 0 \end{cases} \tag{15}$$

IV. To check whether the slave node penetrates the main element's surface.

If $l = n_i\left[t - p(\xi_c,\eta_c) \right] < 0$, it means that the slave node n_s penetrates the main element surface with the contact point $c(\xi_c,\eta_c)$, where n_i is the unit outer normal vector of the main element surface at the contact point:

$$n_i = \frac{\partial p}{\partial \xi}(\xi_c,\eta_c)\times\frac{\partial p}{\partial \eta}(\xi_c,\eta_c) \Big/ \left| \frac{\partial p}{\partial \xi}(\xi_c,\eta_c)\times\frac{\partial p}{\partial \eta}(\xi_c,\eta_c) \right| \tag{16}$$

If $l \geq 0$, it means that the slave node n_i does not penetrate the main element's surface, that is, there is no contact or collision between two objects. Therefore, the processing is completed at the node n_i without any treatment. The search for the relationship between the slave node and the main element's surface will be started from next slave node n_{i+1}, as shown in Figure 5.

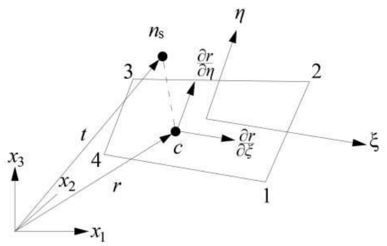

Fig.5 The relationship between the slave node and the main element's surface

V. If the slave node penetrates the main element surface, the normal contact force shall be applied between the slave node n_s and the contact point c.

$$f_s = -k_i n_i \tag{17}$$

Where, k_i is the stiffness factor of the main element surface and meets the following formulas.

$$k_i = fK_i A_i^2 / V_i \tag{18}$$

Where, K_i is the bulk modulus of the contact element; A_i is the area of the main element surface; V_i is the volume of the main element; f is a scale factor of the contact stiffness, and 0.10 is set as the default value. In case of excessive penetration during calculation, keyword controlling parameters could be used to amplify penalty factors. If f>0.4, the calculation may become unstable unless the time step is reduced.

To apply contact force vector f_s in slave node n_s, according to Newton's third law, a force in opposite direction $-f_s$ acts on the contact point c in the main element surface, the contact force at point c could be assigned equivalently to the nodes on the main element according to the following formula:

$$f_{jm} = -\phi_j(\xi_c, \eta_c) f_s, \quad j = 1,2,3,4 \tag{19}$$

Where, f_{jm} is contact force at the nodes, $\phi_j(\xi_c, \eta_c)$ is a two-dimensional shape function in the main element surface, and $\sum_{j=1}^{4} \phi_j(\xi_c, \eta_c) = 1$ at the contact point c.

VI. To calculate the tangential contact friction force

If the normal force of the slave node n_s is f_s, the maximum friction will be $F_Y = \mu |f_s|$ and μ is the friction coefficient. Suppose that the contact friction force of n_s is F^n at the last time (t_n), the friction F^* at the present time (t_{n+1}) may be $F^* = F^n - k\Delta\alpha$, where k is interface stiffness, and $\Delta\alpha = p^{n+1}(\xi_c^{n+1}, \eta_c^{n+1}) - p^{n+1}(\xi_c^n, \eta_c^n)$. The contact friction force at the present time could be fixed by the following formula:

$$F^{n+1} = \begin{cases} F^* & \text{if } |F^*| \le F_Y \\ F_Y F^* / |F^*| & \text{if } |F^*| > F_Y \end{cases} \tag{20}$$

3. Numerical simulation construction of the yielding bolt

3.1 Parameters of the yielding bolt

Table 1 Mechanical parameters of the rod body

Name and Spec.	Yield strength MPa	Tensile strength MPa	Elongation %
MSGLW-500/20, 22(RY)	⩾500	⩾630	⩾15
MSGLW-600/20, 22(RY)	⩾600	⩾750	

The yielding pipe is one of the core components of the yielding bolt. Table 2 shows basic parameters of the yielding pipe in MT146.2-201 series. This paper focuses on the effects of eccentric load on the performance of the

yielding bolt. Therefore, the numerical simulation analysis was carried out by taking the yielding bolt in MSGLW-500/20(RY) series as an example. The yield strength of the material is 500 MPa; The bolt's diameter is 20 mm; The yielding point is 160 kN; The maximum yielding amount is 25 mm, and the yielding pipe height is 40 mm.

Table 2 Basic parameters of the yielding bolt

Materials /MPa	Diameter /mm	Yielding point /kN	Yielding amount /mm	Yielding pipe height /mm
500	20	160	25	40
	22	180		
600	20	180		
	22	220		

3.2 Numerical Model

There are 7 steps in numerical modeling, including the geometric modeling, material selection, mesh generation, contact definition, boundary conditions application, solving and post-processing [13, 14]. During the model modeling, the three-dimensional software SolidWorks was adopted. The commands, such as the rotate and cut etc., were used to create the gasket, the yielding pipe and the tray. Their corresponding positional relations were defined in the component module [15]. Due to the symmetry in the model, half of the model was necessary to reduce the calculated amount [16, 17]. In view of the material selection, the metal material selected a bilinear isotropic elastoplastic constitutive model for the metal gasket, tray and the yielding pipe, while the nylon material chosen the constitutive equation based on the strain energy function for the nylon gasket [18, 19]. To define the contact relations, there were 5 pairs of contact relations among the bolt's components, and there were 2 pairs of contact relations among the components in themselves [20]. In terms of mesh generation, the mesh size is set based on the geometry's curvatures and contact relations. The mesh density is in general higher when the curvature is larger. In the contact relation, the mesh size of the slave surface is smaller than that of the master surface [21]. According to the above principles, the mesh size is set between 0.6 mm and 3.0 mm. A total of 45430 tetrahedral elements and 1296 quadrilateral elements were generated [22]. For boundary conditions, the symmetry constraints were imposed on the symmetry surfaces, and the bottom of the tray was fixed [23]. In solving the model, the generated mesh model was imported into Nastran Solver, and the advanced nonlinear solver was applied. The results were imported into Patran software for post-processing in order to extract various stresses, plastic strains etc.

To simulate the load acted on the yielding pipe due to the tension of the anchor, the rigid plane was added in the numerical modeling. In addition, the reference points for the rigid plane were defined [24]. Then the influence of eccentric load on the prestressed yielding bolt's performance were analyzed by adjusting the rigid plane's angle, as shown in Figure 6. The hole deviation should be generally between 0° and 5°, so the dip of the rigid plane should also be controlled within 5°.

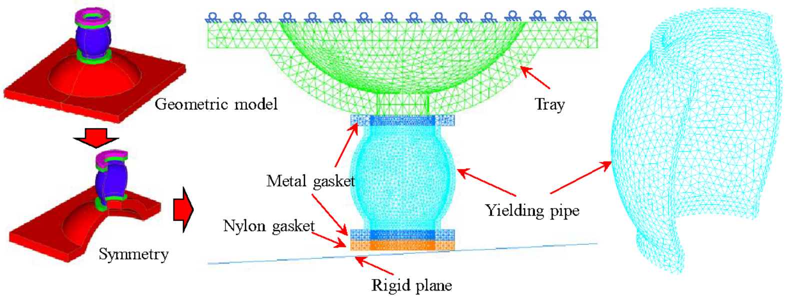

Fig.6 The numerical model

4. Analysis of the influence of eccentric load on the yielding pipe's performance

The yielding pipe's performance could be reflected through its displacement-load relations, use efficiency, plastic strain of its components and the absorbed energy. This chapter mainly analyzes influence of eccentric load on the 4 indicators.

4.1The influence of eccentric load on displacement-load relations

The displacement-load relations could be used to get the yielding bolt's yielding point, amounts etc. These parameters could be used to choose proper prestressed yielding bolts. The displacement-load relations could be extracted by the post-processing function of Patran software, as shown in Figure 7.0

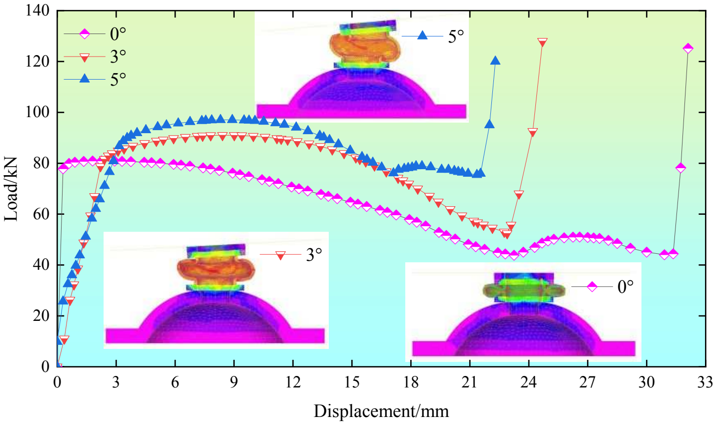

Fig.7 The displacement-load relations under various eccentric load

Figure 7 indicates that:

(1) When the eccentric loads are 0°, 3° and 5°, the yielding points of half of the yielding pipe are 80.9 kN, 90.1 kN and 97.0 kN, respectively. Therefore, the corresponding yielding points of the whole pipe are 161.8 kN, 180.2 kN and 194 kN, and the corresponding displacement of the yielding points are 1.42 mm, 7.81 mm and 8.96 mm, respectively.

(2) As the eccentric load increases, the pipe's yielding point and the corresponding displacement also increases. This could be explained by the following Figures. In Figure 8 (a), when the eccentric load is 0°, after the extrusion by the rigid plane, the pipe's whole stress distribution is even, and there are loads on the integral material. In Figures 8 (b) and 8 (c), when the eccentric loads are 3° and 5°, the pipe's stress spreads from one side to the whole, and the displacement of the yielding point increases, which is due to the change of the pipe's yielding route resulted from the eccentric load.

(3) When the eccentric loads are 0°, 3° and 5°, the pipe's yielding amounts are 31.2 mm, 23.5 mm and 21 mm, respectively. The yielding amount increases with the eccentric load.

In order to further explore the relations among eccentric load, the yielding point and its corresponding displacement as well as the yielding amount, these parameters within the range of 5° were recorded, and their functional relationships were also given, as shown in table 3.

Table 3 Influence of eccentric loads on key parameters of the yielding bolt

Eccentric load θ/°	Yielding point F_{max}/kN	Yielding point's displacement L_1/mm	Yielding amount L_2/mm
0	161.8	1.4	31.2
1	166.2	4.2	28.0
2	174.4	6.0	25.3
3	180.2	7.8	23.5

4	190.0	8.2	22.0
5	194.0	8.96	21.0
Fitting relation	F_{max}=6.8058θ+160.752	L_1=1.4717θ+2.419	L_2=-2.0229θ+30.224
Correlation coefficient	R^2=0.9895	R^2=0.9237	R^2=0.9597

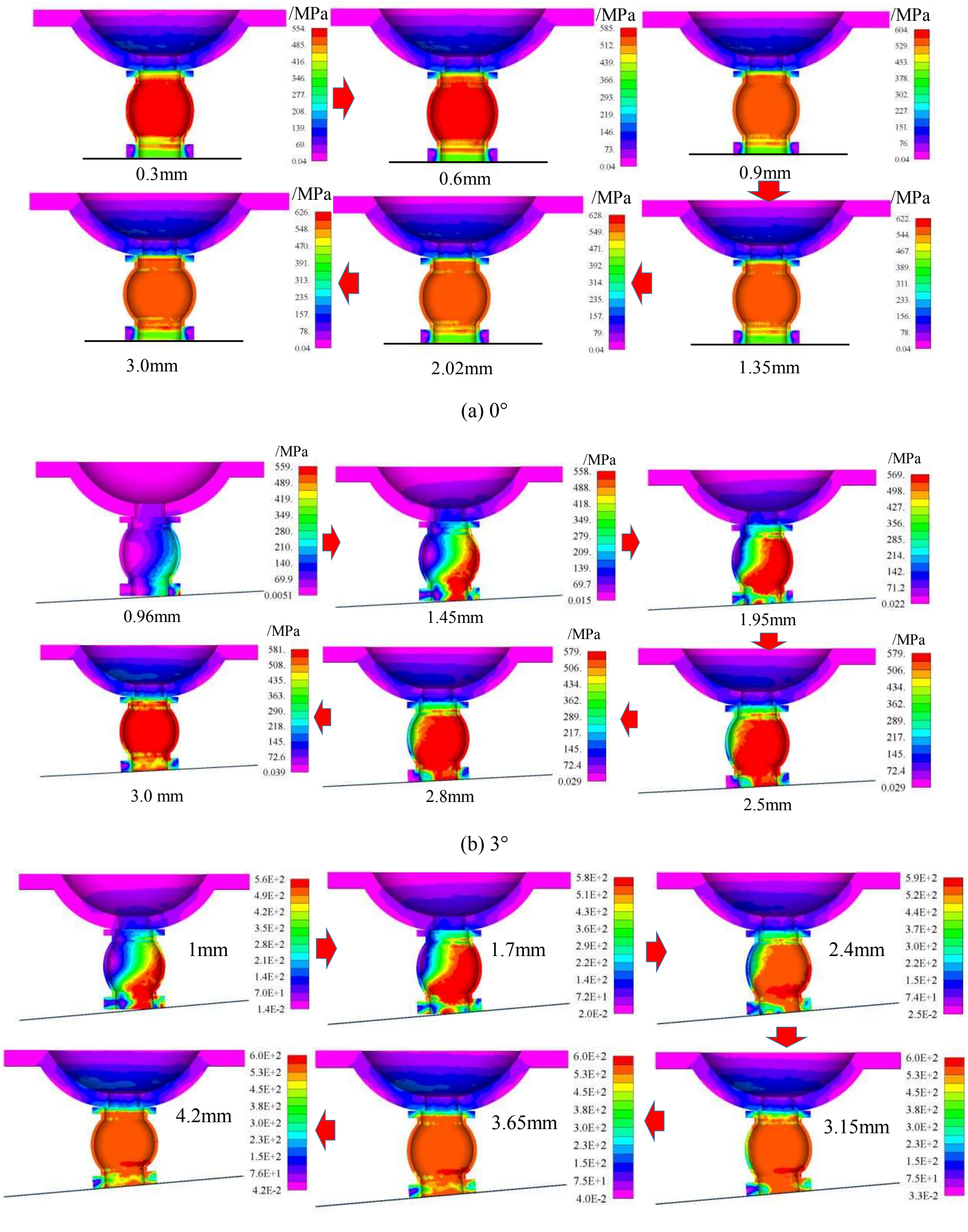

Fig.8 Mises stress distribution characteristics of yielding bolt components

4.2 The use efficiency of the yielding pipe material

The use efficiency of the yielding pipe material could be reflected whether it can bear loads evenly, and the loads can be reflected through the pipe's Mises stress distribution characteristics. According to Chapter 4.1, the Mises stress concentration occurred at the stage of the initial displacement under eccentric load, while the even distribution of the Mises stress occurred at the stage of the later displacement. Therefore, Mises stresses were

extracted when the deformation amounts were 2 mm and 10 mm.

Figure 9 (a) shows the Mises stress distribution laws under various eccentric loading conditions when the deformation is 2 mm. To quantitatively analyze the distribution characteristics, the yielding pipe's 7995 tetrahedral elements' Mises stresses were given, as shown in Figure 9 (b).

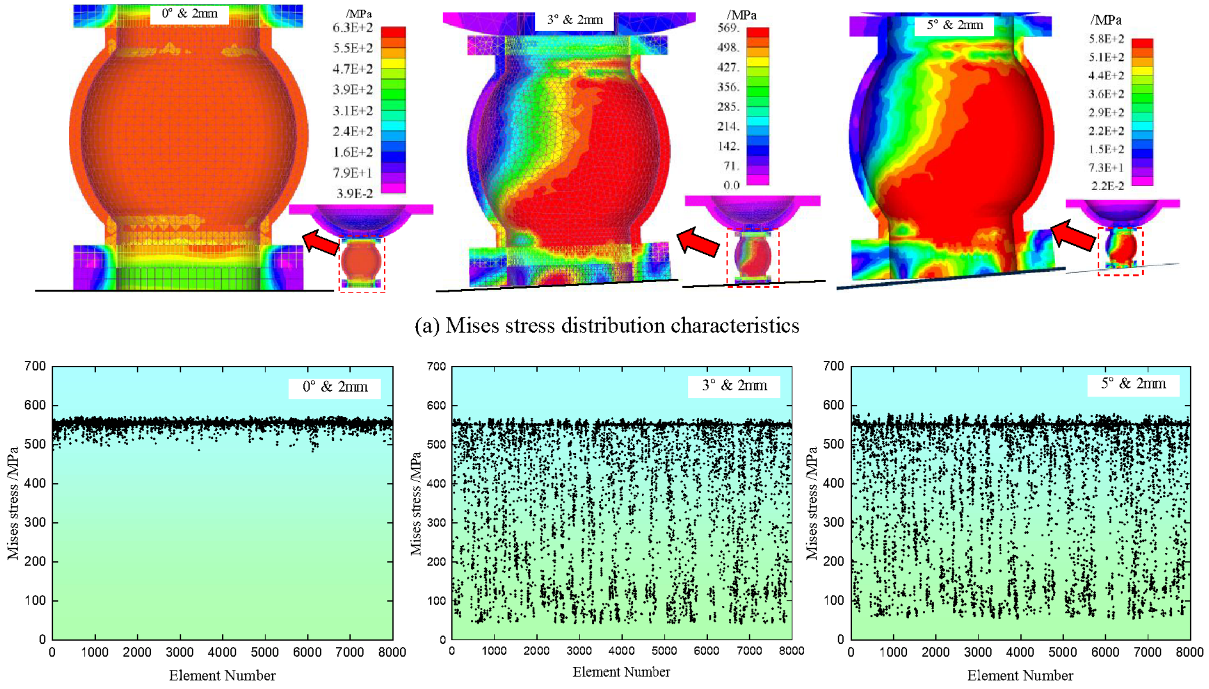

(a) Mises stress distribution characteristics

(b) Element's Mises stress

Fig.9 Mises stress at the displacement of 2 mm

Figure 9 (a) indicates that when the eccentric load is 0°, Mises stresses distribute evenly within the yielding pipe. When the eccentric loads are 3° and 5°, Mises stresses concentrate at the right side of the yielding pipe. Therefore, eccentric load could affect the pipe's use efficiency at the stage of initial deformation. Figure 9 (b) shows that when the eccentric load is 0°, Mises stresses concentrate between 500 and 550 MPa, while they concentrate between 50 and 550 MPa when the eccentric loads are 3° and 5°, further illustrating that eccentric load have impacts on the pipe's use efficiency at the state of initial deformation.

Similarly, Figure 10 (a) shows Mises stress distribution under different eccentric load when the displacement is 10 mm. Figure 10 (b) gives the yielding pipe's 7995 tetrahedral elements' Mises stress values.

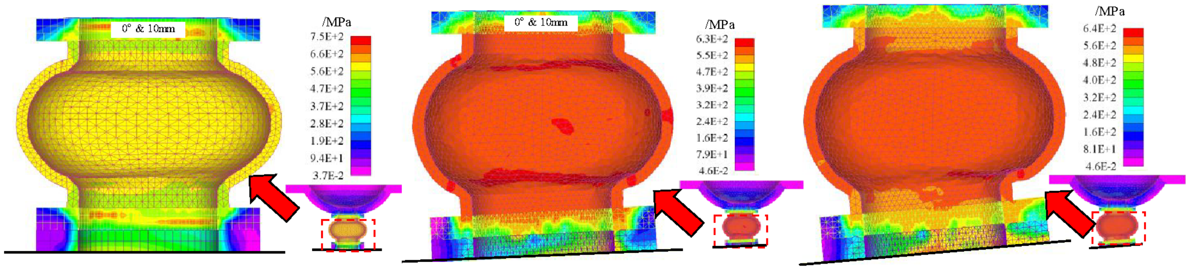

(a) Mises stress distribution characteristics

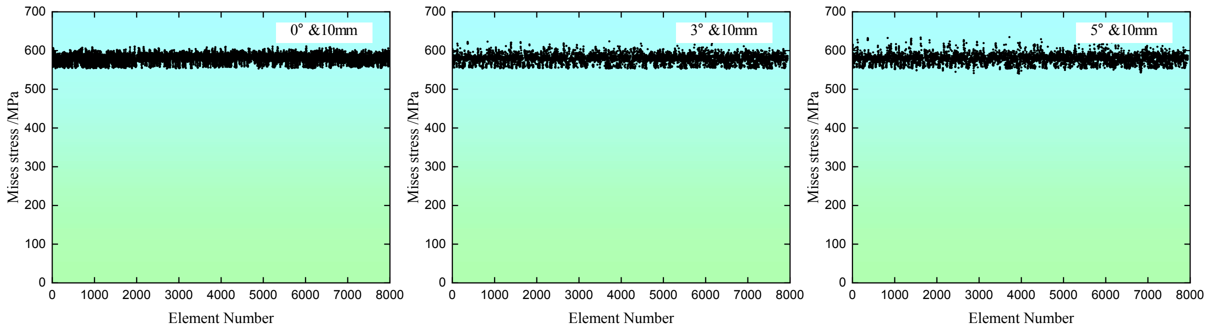

(b) Element's Mises stress

Fig.10 Mises stresses with the deformation of 10 mm

In Figure 10 (a), Mises stresses distribute evenly under various eccentric load, indicating that the use efficiency of the yielding pipe is higher at the stage of later deformation. In Figure 10 (b), Mises stresses concentrate between 500 MPa and 550 MPa, further indicating that eccentric load have fewer impacts on the use efficiency of the yielding pipe material.

To further analyze the use efficiency, the elements' Mises stresses were constantly extracted and the data were exported after the meshing of the finite model, as shown in Figure 11.

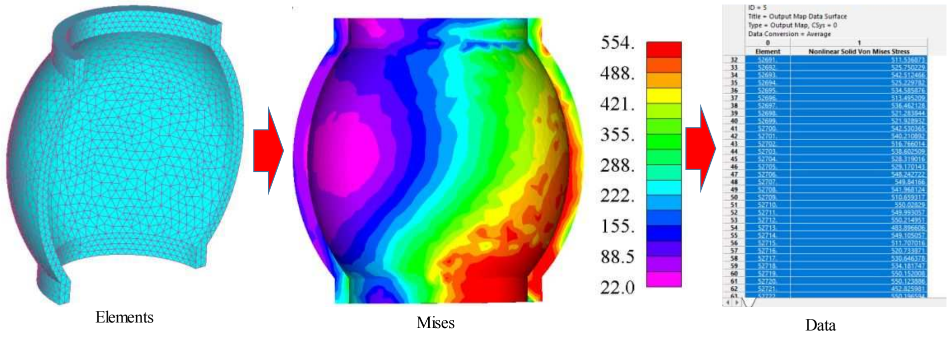

Fig.11 Mises stresses of the yielding pipe

C++ program was adopted to realize the extraction process of the Mises stress data. 7995 tetrahedron elements' Mises stresses were recorded and their relative average deviations were given under different displacement, as shown in Figure 12.

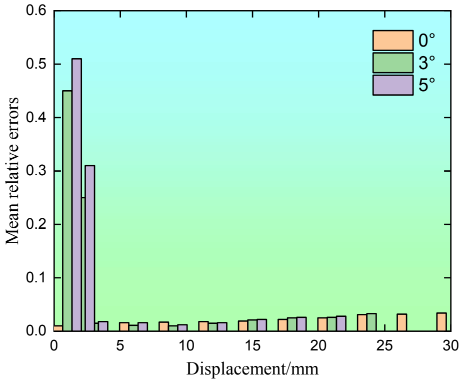

Fig.12 Mises stresses of the yielding pipe

Figure 12 indicates that:

(1) When eccentric loads are 3° and 5°, and the displacement is between 0 and 2 mm, the mean relative errors of the Mises stresses are relatively large, the stresses are uneven in the pipe and the use efficiency is low. When the displacement is between 5 and 24 mm, the mean relative errors are within 4%, the stresses are uniformly distributed and the use efficiency is high.

(2) When the eccentric load is 0°, the mean relative errors of Mises stresses are within 4%, the stresses are uniform and the materials are used effectively.

The analyses indicate that eccentric load have significant influences on the use efficiency of the yielding pipe material at the stage of initial displacement, while they almost have no effects at the stage of the later displacement.

4.3 Plastic properties of bolt's components

During the yielding process of the prestressed yielding bolt, it is necessary to occur plastic failure for the yielding pipe. Meanwhile, other components should be at the elastic stage. This section will analyze influence laws of eccentric load on the plastic properties of the yielding bolt's components. As shown in Figure 13, the plastic strains of the yielding pipe were extracted at the displacement of 2 mm.

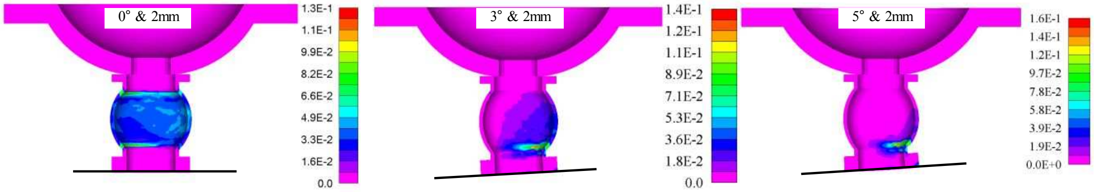

(a) Distribution characteristics of the plastic strain

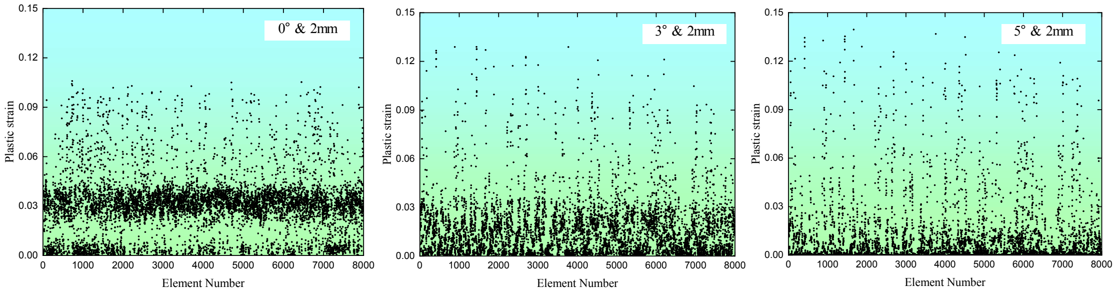

(b) The element's plastic strains

Fig.13 Plastic strains at the displacement of 2 mm

Figure 13 shows that the plastic strains of the bolt's components concentrate in the yielding pipe under different eccentric loads, while other components are basically at the elastic stage. When the eccentric load is 0°, the plastic zone in the yielding pipe is even. When eccentric loads are 3° and 5°, the plastic zone concentrates on the right side of the pipe at the stage of the initial displacement.

The plastic strains with different displacement were extracted for further analysis, as shown in Figure 14.

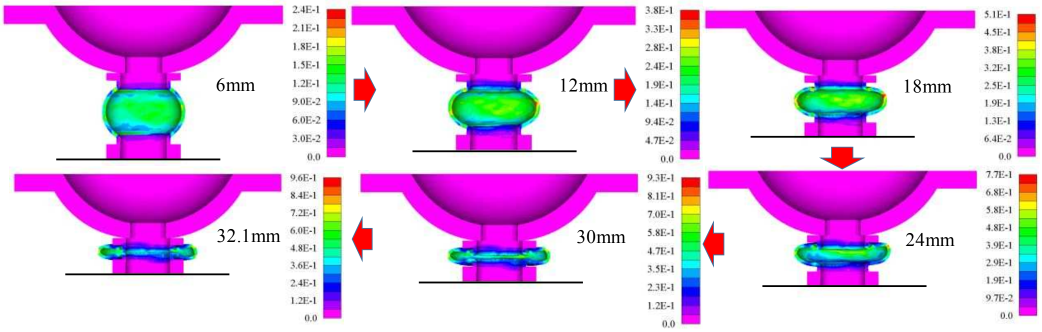

(a) 0°

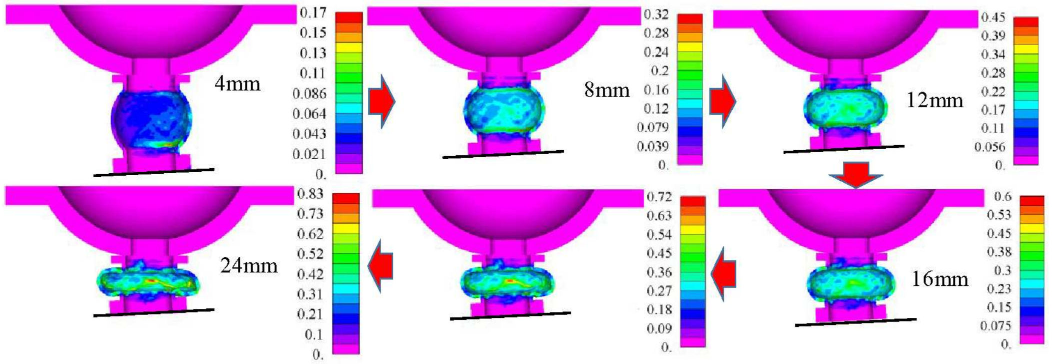

(b) 3°

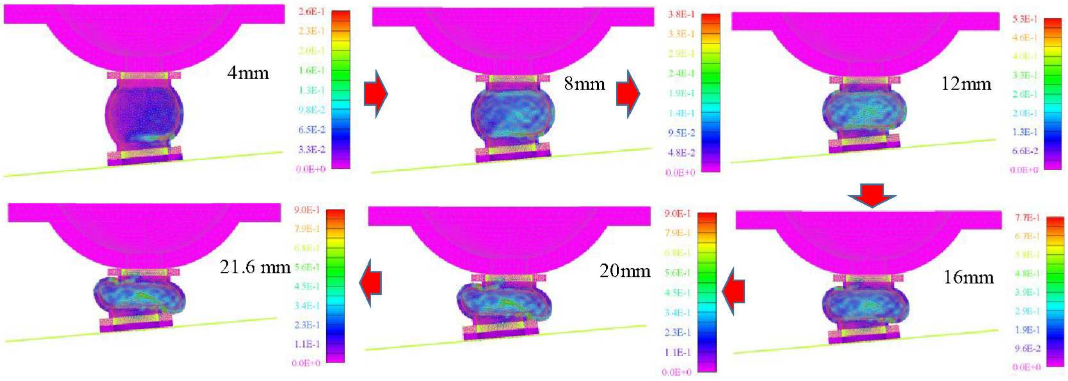

(c) 5°

Fig.14 Plastic strains of the bolt components

Figure 14 shows that Mises stresses of the bolt components concentrate in the yielding pipe with various displacement, while other components are basically at the elastic stage. It could be found out that the plastic strain distribution characteristics are greatly affected by eccentric load at the stage of the initial displacement, while they mainly concentrate in the middle of the yielding pipe at the stage of later displacement.

Above all, eccentric loads could influence the bolt components' plastic strain distribution characteristics at the stage of the initial displacement, while they have a negligible effect at the stage of later displacement.

4.4 Absorptive capacity of the yielding pipe

In the high stress and large deformation roadway, the surrounding rock deformation will release certain energy and the yielding pipe shall absorb part of the deformation energy. The amount and rate of energy absorbed could reflect the performance of the prestressed yielding bolt. As shown in Figure 15, C++ programing was used to add the absorbed elastic energy and plastic energy together within the element to get the energy absorbed by the yielding pipe.

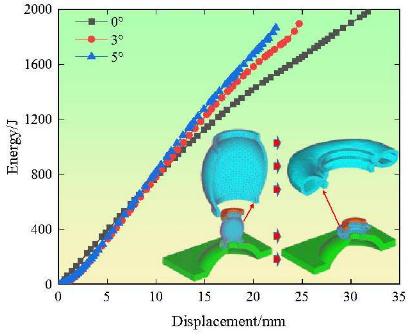

Fig.15 Law of the absorbed energy by the yielding pipe with various eccentric loads

Figure 15 indicates that:

(1) During the yielding process, when eccentric loads are 0°, 3° and 5°, the energy absorbed by half of the yielding pipe is 2021 J, 1894 J and 1863 J, respectively. Thus, the energy absorbed by the whole yielding pipe is 4042J, 3788 J and 3726 J, respectively. As the eccentric load increases, the absorbed energy will reduce, and the extent is not distinct and is basically within 10%.

(2) At the stage of the initial displacement, when the eccentric load is 0°, the absorbed energy by the yielding pipe is larger than that when eccentric loads are 3° and 5°. While it is the opposite at the stage of the later displacement.

(3) The rate of energy absorbed are basically stable with various eccentric loading conditions, thus proving that the yielding pipe's structure is stable during the yielding process.

5 Discussions

According to the analyses in Chapter 4, eccentric load could affect the use efficiency of the yielding pipe, plastic deformation of the bolt components as well as the yielding pipe's absorption rate at the stage of the initial deformation. However, these impacts can be neglected at the stage of the later deformation. At this stage, eccentric loads mainly affect the yielding bolt's displacement-load relations which are mainly reflected by the yielding point and its corresponding displacement as well as the yielding amount. This was analyzed by a specific case in the following.

Large deformation of the roadway occurred during the mining of the working face in Tiefa Coal Mine. The yielding bolts were used in order to solve the problem. The yielding point was larger than 150 kN and the yielding amount was larger than 25 mm. According to table 2, the yielding bolt in MSGLW-500/20(RY) series was selected with the yielding point of 160 kN and the yielding amount of 25 mm, which could meet the design requirements. However, the on-site borehole deviation was between 3° and 5°. According to the analysis in Chapter 4, when the borehole deviation was generally between 3 and 5°, the yielding point should be between 180.2 and 194.0 kN, and the yielding amount should be between 21.0 and 23.5 mm. Thus, the yielding amount could not satisfy the design requirement due to the borehole deviation.

In the field, the borehole deviation was adjusted to control the yielding amount and point. According to influence laws of eccentric loads on the yielding bolt's displacement-load relations, the following relationship existed between the yielding amount and eccentric loads: L_2=-2.0229θ+30.224. Therefore, the eccentric load was controlled between 0 and 2.5°. At this point, the MSGLW-500/20(RY) yielding bolt's yielding point was between 160.75 and 177.76 kN, and the yielding amount was between 25.2 and 30.2 mm, which could meet the design requirement.

6 Conclusions

In this paper, the yielding bolt's numerical analysis model was firstly established by the numerical simulation method. Then the impacts of eccentric load on displacement-load relations, use efficiency of the yielding pipe, plastic strains of bolt components as well as the absorptive capacity of the yielding pipe were analyzed in detail. The conclusions are as follows:

(1) Eccentric loads affected use efficiency of the yielding pipe, plastic strains of bolt components and the absorptive capacity to a certain degree at the stage of initial displacement, while these impacts could be neglected at the stage of the later deformation.

(2) Eccentric loads mainly affected the displacement-load relations of the yielding bolt. As the eccentric load increased, the yielding point and its corresponding displacement increased linearly, while the yielding amount decreased linearly.

(3) In the field, eccentric loads could be adjusted to control the yielding point and amount of the yielding bolt in order to meet the design requirement of roadway support.

Ethics statement: The article is about mining science and does not involve ethics.

Data Availability: Data available from the Dryad Digital Repository: https:// doi:10.5061/dryad.34tmpg4gg [25]. Dryad review URL: https://datadryad.org/stash/share/TD5tUaCfaCiJYAlxXq1Yi34CpcOuhIaE9fXXH01M9Zo.

Authors' Contributions: Y.T. participated in the design of the study and drafted the manuscript; H.X. carried out the statistical analyses; S.H. collected field data; J.M. conceived of the study and designed the study; W.L. coordinated the study and helped draft the manuscript. All authors gave final approval for publication.

Competing Interests: The authors declare no competing financial interests.

Funding statement: This work was supported by LiaoNing Revitalization Talents Program (XLYC1807219).

Permission to carry out fieldwork: We obtained the appropriate permissions and licenses to conduct the fieldwork detailed in our study.

Acknowledgements: This work was supported by LiaoNing Revitalization Talents Program (XLYC1807219). The authors gratefully acknowledge the financial support from the organization.

References

[1] Majcherczyk, T., Niedbalski, Z., Malkowski, P. & Bednarek, L. 2014 ANALYSIS OF YIELDING STEEL ARCH SUPPORT WITH ROCK BOLTS IN MINE ROADWAYS STABILITY ASPECT. *Archives of Mining Sciences* **59**, 641-654. (doi:10.2478/amsc-2014-0045).

[2] Wu, X., Jiang, Y., Wang, G., Gong, B., Guan, Z. & Deng, T. 2019 Performance of a New Yielding Rock Bolt Under Pull and Shear Loading Conditions. *Rock Mechanics and Rock Engineering* **52**, 3401-3412. (doi:10.1007/s00603-019-01779-8).

[3] Wu, C., Wang, M., Dong, Y., Cheng, Z. & Meng, H. 2015 Growth, bolting and yield of garlic (Allium sativum L.) in response to clove chilling treatment. *Scientia Horticulturae* **194**, 43-52. (doi:10.1016/j.scienta.2015.07.018).

[4] Sun, X., Wang, L., Lu, Y., Jiang, B., Li, Z. & Zhang, J. 2018 A yielding bolt - grouting support design for a soft-rock roadway under high stress: a case study of the Yuandian No. 2 coal mine in China. *Journal of the Southern African Institute of Mining and Metallurgy* **118**, 71-82. (doi:10.17159/2411-9717/2018/v118n1a9).

[5] Xiang, L., Xiao, M. & Meng, P. Analysis of Strength and Deformation of Sand-grained Materials of Earth Dam Foundation under K0-consolidation Condition. *Water Resources and Power* **34**, 141-145.

[6] ZHU, A.-l., ZHANG, Y., AI, M.-l. & XU, J.-q. 2017 Reinforcement mechanism of slopes with yielding anchor cables based on numerical simulation of FLAC3D *Chinese Journal of Geotechnical Engineering* **39**, 713-719.

[7] YANG, Y. 2017 Study of Performance and Application of Yielding Anchor Bolt with Large Allowable Deformation. *Tunnel and Construction* **37**, 321-329.

[8] LIAN, C.-j. & WANG, G. 2013 Influence of design parameters of prestressed yieldable bolts on stability of deep mine roadway. *Chinese Journal of Geotechnical Engineering* **34**, 452-458.

[9] Shan, R., Yang, H., Zhou, H. & Tao, Y. 2014 Design of support parameters and energy constitutive model for yielding anchor bolt. *Journal of China University of Mining and Technology* **43**, 241-247.

[10] Yang, F. 2009 Research on Deformation and Failure Character and Yielding Support Mechanism in High Stress Soft Rock Roadway. Xuzhou, China university of mining and technology.

[11] GUO, Y., JIANG, F., ZHOU, H. & XIANG, X. 2015 The mechanism of the new-type yielding bolt. *Mechanics in Engineering* **37**, 193-196.

[12] ZHANG, X., Lianguo, W., WU, Y. & DONG, X. 2008 Numerical Simulation of Support with High-Strength Yielding Bolt. *Journal of Mining & Safety Engineering* **25**, 46-49.

[13] El-Behery, S.M., El-Askary, W.A., Hamed, M.H. & Ibrahim, K.A. 2012 Numerical and experimental studies of heat transfer in particle-laden gas flows through a vertical riser. *International Journal of Heat and Fluid Flow* **33**, 118-130. (doi:10.1016/j.ijheatfluidflow.2011.09.011).

[14] Ziadé, P., Feero, M.A. & Sullivan, P.E. 2018 A numerical study on the influence of cavity shape on synthetic jet performance. *International Journal of Heat and Fluid Flow* **74**, 187-197. (doi:10.1016/j.ijheatfluidflow.2018.10.001).

[15] Liu, B., Xu, T., Xu, X., Wang, Y., Sun, Y. & Li, Y. 2016 Energy absorption mechanism of polyvinyl butyral laminated windshield

subjected to head impact: Experiment and numerical simulations. *International Journal of Impact Engineering* **90**, 26-36. (doi:10.1016/j.ijimpeng.2015.11.010).

[16] Al Kajbaf, A., Fanaie, N. & Faraji Najarkolaie, K. 2018 Numerical simulation of failure in steel posttensioned connections under cyclic loading. *Eng Fail Anal* **91**, 35-57. (doi:10.1016/j.engfailanal.2018.04.024).

[17] Binar, T., Švarc, J., Vyroubal, P., Kazda, T., Rolc, S., Dvořák, A. & Dostál, P. 2018 The use of numerical simulation for the evaluation of special transparent glass resistance. *Eng Fail Anal* **91**, 433-448. (doi:10.1016/j.engfailanal.2018.04.014).

[18] Chen, Z., Li, X., Yang, Y., Zhao, S. & Fu, Z. 2018 Experimental and numerical investigation of the effect of temperature patterns on behavior of large scale silo. *Eng Fail Anal* **91**, 543-553. (doi:10.1016/j.engfailanal.2018.04.043).

[19] Esmaeili-Goldarag, F., Babaei, A. & Jafarzadeh, H. 2018 An experimental and numerical investigation of clamping force variation in simple bolted and hybrid (bolted-bonded) double lap joints due to applied longitudinal loads. *Eng Fail Anal* **91**, 327-340. (doi:10.1016/j.engfailanal.2018.04.047).

[20] D'Albano, G.G.S., Lukas, T., Su, F., Korakianitis, T. & Munjiza, A. 2017 Numerical comparison of some contact detection algorithms. *Engineering Computations* **34**, 832-851. (doi:10.1108/ec-01-2016-0047).

[21] Ma, S., Zhao, Z., Nie, W. & Gui, Y. 2016 A numerical model of fully grouted bolts considering the tri-linear shear bond–slip model. *Tunnelling and Underground Space Technology* **54**, 73-80. (doi:10.1016/j.tust.2016.01.033).

[22] Zou, Y.S., Zhang, S.C., Ma, X.F., Zhou, T. & Zeng, B. 2016 Numerical investigation of hydraulic fracture network propagation in naturally fractured shale formations. *J Struct Geol* **84**, 1-13. (doi:10.1016/j.jsg.2016.01.004).

[23] Ha, S. 2009 Numerical Simulations of Crack Initiation and PropagationUsing Cohesive Zone Elements. *Journal of the Computational Structural Engineering Institute of Korea* **22**, 519-526.

[24] Johnson, S.M., Williams, J.R. & Cook, B.K. 2008 Quaternion-based rigid body rotation integration algorithms for use in particle methods. *International Journal for Numerical Methods in Engineering* **74**, 1303-1313. (doi:10.1002/nme.2210).

[25] Tai, Yang (2020), The Influence Law of Eccentric Load on the Performance of Yielding Bolt, Dryad, Dataset, https://doi.org/10.5061/dryad.fqz612jpt.

Appendix B

The Influence Law of Eccentric Load on the Performance of Yielding Bolt

Yang Tai [1], Hongchun Xia [2, *], Shaoping Huang [3, *], Jie Meng [4], Wei Li [2]

1. School of Mines, China University of Mining and Technology, Xuzhou 221116, China;

2. College of Civil Engineering and Architecture, Dalian University, Liaoning, Dalian 116622, China;

3. Faculty of Engineering, China University of Geosciences, Wuhan, Hubei 430074, China;

4. Department of Civil Engineering, University of Ottawa, 161 Colonel by, Ottawa (Ontario) K1N 6N5, Canada.

* Correspondence author: cumtcqty@gmail.com

Abstract: In order to adapt high stress and large deformation of roadways, the prestressed yielding bolt has been developed. However, borehole deviation makes the boreholes out of perpendicular to the roadway surface, and the prestressed yielding bolt will be under eccentric load. In order to reveal the influence law of eccentric load on the performance of the prestressed yielding bolt, the numerical simulation was carried out in this paper. Thus, the influence of eccentric load on displacement-load relations, use efficiency for the yielding pipe material, plastic strains of the bolt components, as well as the absorptive capacity of the yielding pipe were analyzed. The results are as follows: (1). Eccentric loads affected use efficiency of the yielding pipe material, plastic strains of bolt components and the absorptive capacity to a certain degree at the stage of initial displacement, while these impacts could be neglected at the stage of the later displacement; (2) Eccentric load mainly affected the yielding pipe's displacement-load relations. As the eccentric load increased, the yielding point and its corresponding displacement increased linearly while the yielding amount decreased linearly; (3) The eccentric load could be adjusted to control the yielding point and amount in order to meet the roadway support's requirement for the yielding bolt.

Keywords: Yielding bolt; eccentric load; performance; numerical simulation

1 Introduction

With the advantages of sound control effects, low costs and simple operation, the bolt is one of the most common support methods for surrounding rocks in coal mines [1, 2]. As the mining depth of coal mines increases, the roadway surrounding rocks are characterized with high stress and large deformation [3]. At this time, common bolts are difficult to meet the support requirements, so the structures of bolts have been optimized. Moreover, various types of extensible bolts have also been design. Among them, the most common type is the yielding bolt developed by American Jennmar Corporation, as shown in Figure 1 (a). It could be seen that the yielding bolt consists of nuts, the yielding pipe, tray and high-intensity rod body. As one of the core components, the yielding pipe is made of seamless steel pipe. The excessive deformation energy could be released through the deformation of the yielding pipe. After the yielding, the yielding bolt has stronger constraint forces and could effectively control the roadway surrounding rock deformation and ensure the stability of the roadway [4].

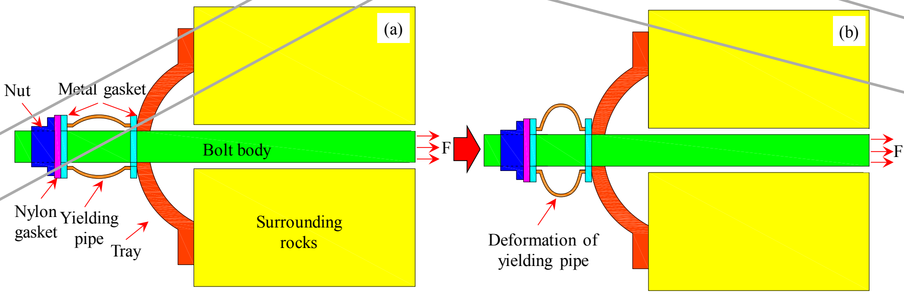

Fig.1 The structure of the yielding bolt

Before installation of the yielding bolt, professional drilling equipment should be used to drill holes with certain depths and diameters. Due to equipment accuracy and personal operations, it is inevitable to occur hole deviations

during the drilling process, as shown in Figure 2. In this case, the yielding bolt will ~~occur~~ eccentric load.

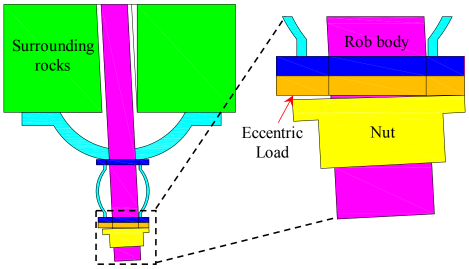

Fig.2 The eccentric load for yielding bolt

In-depth studies have been made on the yielding bolt by scholars both at ~~home and abroad,~~ mainly focusing on evaluation of the yielding effects, selection of key parameters of the yielding bolt, and coupling relationships between the yielding bolt and the surrounding rock. **~~(1) The~~ evaluation of the yielding effects.** Xiang et al. [5] established a mechanical model of the yielding bolt. The finite element shear strength reduction method was used to analyze and evaluate the support effects of the high-intensity yielding bolt; Zhu et al. [6] built a constitutive model of the yielding anchor. Then, FLAC^{3D} software was used to analyze the slope reinforcement effects of the anchor; Yang [7] introduced the structure and the support process of the large-scale yielding bolt, and analyzed its performance characteristics at the stages of elastic deformation, sliding and yielding by ANSYS software. Meanwhile, the yielding results were also evaluated; Lian et al. [8] built a 3D mechanical model with the consideration of the shear failure between the prestressed yielding bolt and the rock, and developed the corresponding calculation method. **(2) The selection key parameters of the yielding bolt.** Shan et al. [9] divided the tensile process of the yielding bolt into the elastic stage, the yielding stage and the plastic hardening stage. Based on the energy constitutive equations at different stages, the ultimate load calculation formula was given; Yang [10] discussed various yielding bolts' basic mechanical parameters such as the yielding point, yielding load stability, the maximum yielding amount etc., which could provide basis for the selection of the yielding bolt. **(3) The coupling relationships between the yielding bolt and the surrounding rock.** Guo et al. [11] introduced a new type of pressure-dispersive yielding bolt. Its mechanism and mechanical behaviors were analyzed, and the coupling relationships between the yielding bolt and the surrounding rock were given; Zhang et al. [12] systematically analyzed variations of surrounding rock stresses, displacements and axial forces before and after the support of the yielding bolt.

The studies mainly focus on evaluation of the yielding effects, the key parameters of the yielding bolt as well as coupling relationships between the yielding bolt and the surrounding rock. However, the influence laws of eccentric load on the performance of the yielding bolt have not been studied. In this paper, the numerical model of the yielding bolt was established. Then the influence laws of eccentric loads on the performance of the yielding bolt were explored. At the same time, the engineering design was carried out.

2 Numerical calculation method

2.1 Calculation method

2.1.1 Method of solving nonlinear equations

During the yielding process of the yielding bolt, multiple nonlinearities are involved, such as the geometric nonlinearity, material nonlinearity, contact nonlinearity etc. In addition, the bolt's structure is complex. Therefore, the traditional theoretical calculations could not reveal influence laws of eccentric load on the performance of the yielding bolt. Thus, the numerical simulation was adopted, whose basic flow is shown in Figure 3.

Notes

3-1 Adapt what exactly?
Adapt is the incorrect choice of words in this context.

3-2 The logic does not follow on from previous sentence, that is “however” is incorrectly used here.

3-3 What borehole? What connection is there between borehole and stress/deformation?

3-4 This makes no sense.

3-5 Why is this so?

3-6 I have not heard of these laws. Are the authors really intending on revealing the influence or determine/calculate etc?

3-8 I think then authors mean “a simulation.” There could/can be others.

3-9 Were the simulations carried out in the paper or, are the results of the modelling and subsequent analysis presented in the paper?

3-10 I am unfamiliar with this term, what is meant by this term?

3-11 Is the ‘pipe material’ yielding or, the ‘yield pipe’ is some sort of material that can have some sort of efficiency?

3-12 Again, I am unfamiliar with the term “use efficiency.” What is it supposed to mean?

3-13 This is a statement rather than a conclusion...’to a certain degree’ of what - relax, are near zero etc?

3-14 What impacts? What ‘later displacement?

3-16 What are “sound control effects?’

3-17 How is a something like a bolt which is an entity, an operation or process simple or otherwise?

3-18 What “bolt” are the authors referring to - some type of mechanical screw or, the specific type of support mechanism used in ground control of which there are two types - rockbolts and cable bolts. Are the authors referring to both these types of ground support bolts or to one or the other?

3-20 in the surrounding rockmass

3-21 Does the roadway have “high stress” or is the rockmass subjected to high stress?

3-22 What is “this time?” Or do the authors mean “Currently, common bolts...”

3-23 What are “common bolts?” How do they differ from say “rare” or “complex” bolts?

3-25 In what way are they “difficult?” Do the author mean they are unable to meet the service requirements need for ground control?

Notes

3-26 I am unfamiliar with what and how “the structures” have been “optimised”.

3-27 What are “extensible bolts?”

3-28 Background information about this rockbolt should be referenced by the authors.

3-29 Can

3-32 A nut

3-33 “rod body” does not appear in Figure 1a. Do the authors mean “bolt body?”

3-34 Why is the “yielding pipe” more of a core compenent compared to the other components? What are core and which are non-core?

3-35 What is “deformation energy?’ What is its source? Under what circumstances does it become “excessive?”

3-37 What are “constraint forces?’ Why does yielding lead to higher constrain forces?”

3-38 How can “constraint forces” lead to effective control or an underground roadway?

3-39 I cannot appreciate how yielding of a bol,t leads to better stability.

3-41 Is required

3-42 of a given diameter, to a certain prescribed depth and at designed inclination.

3-43 Professional as opposed to non-professional?

3-44 Isn’t (in)accuracy an outcome rather than a determining factor? What are “personal operations?”
I think there are other factors also that result in deviation from the designed drill pattern.

4-1 What does this mean?

4-2 Where exactly is “home” and “abroad?” It is relative - the authors backyard, neighbourhood, province, region or do they mean country?

4-3 With respect to the evaluation of the yielding effect, Xiang et al [5]...

Appendix C

Cover letter

Dear editor

Thank you for giving me the chance to revise my manuscript. All revisions are marked with red and blue fonts in the article. If you have any questions, please contact me.

Yours sincerely,

Yang Tai
E-mail: cumtcqty@163.com

The Revision for Comments

Reviewer: 1

1. Among them, the most common type is the yielding bolt developed by American Jennmar Corporation, as shown in Figure 1.

Thank you for your comments. To date, various types of yielding bolts have been designed, among which, the pre-stressed yielding bolt is most popular one (developed by the Jennmar Corporation, as shown in Figure 1).

2. Please explain why you used Newton-Raphson method?

Thank you for your comments. There is usually no formula for roots of nonlinear equation, so it is very difficult, even impossible, to find the approximate root of equation. Newton iterative method is one of the important methods to find the roots of the equation. Its biggest advantage is that it has the square convergence near the single root of the equation. In addition, this method is widely used in computer programming.

3. Please explain why is appropriate to use full Newton-Raphson method.

Thank you for your comments. The Newton Raphson method (also named full Newton Raphson method) is suitable for solving the approximate solution of the roots of nonlinear equations, while *Eqn*. (6) is a typical nonlinear equation, so it is recommended to use the full Newton Raphson method.

4. There are 7 steps in numerical modelling

Thank you for your comments. The sentence has been modified to ‘There are seven steps in numerical modelling’.

5. According to the analyses in Chapter 4,

Thank you for your comments. The sentence has been modified to ‘According to the analysis in Section 4’.

6. Where is Tiefa Coal Mine?

Thank you for your comments. Tiefa Mining Area is located in Diaobingshan city, Liaoning province, China

7. The yielding bolts were used in order to solve the problem.

Thank you for your comments. The sentence has been modified to 'The yielding bolts were used in order to solve tackle the problem.'.

8. According to the analysis in Chapter 4,

Thank you for your comments. The sentence has been modified to 'According to the analysis in Section 4'.

9. In this paper, the yielding bolt' s numerical analysis model was firstly established by the numerical simulation method.

Thank you for your comments. The sentence has been modified to 'In this paper, yielding bolt's numerical analysis model was firstly established by a numerical simulation method.'.

10. Eccentric loads affected use efficiency of the yielding pipe,

Thank you for your comments. The sentence has been modified to 'Eccentric loads effected utilization rate of the yielding pipe'.

11. The paper requires a major literature review, which hasn't been done properly.

Thank you for your comments. The introduction has been improved as follows:

Further studies have been made on the yielding bolt, mainly focusing on evaluation of the yielding effects, selecting key parameters of the yielding bolt, and coupling relationships between the yielding bolt and the surrounding rock. (1) With respect to the evaluation of the yielding effect, Xiang et al. [9] established a mechanical model of the yielding bolt. The finite element shear strength reduction method was used to analyse and evaluate the support effects of the high-intensity yielding bolt; Zhu et al. [10] built a constitutive model of the yielding anchor. Then, the FLAC^{3D} software was used to analyse the slope reinforcement effects of the anchor; Yang [11] introduced the structure and the support procedure of the large-scale yielding bolt. Besides, its performance at the stages of elastic deformation, sliding and yielding were analysed by ANSYS software. Meanwhile, the yielding results were also evaluated; Lian et al. [12] built a 3D mechanical model with the consideration of the shear failure between the prestressed yielding bolt and the rock. Based on that, a corresponding calculation method was developed. (2) With respect to key parameters selection of the yielding bolt, Shan et al. [13] divided the tensile process of the yielding bolt into the elastic stage, the yielding stage and the plastic hardening stage. Based on the energy constitutive equations at different stages, the ultimate load calculation formula was given; Yang [14] discussed various yielding bolts' basic mechanical parameters such as the yielding point, yielding load stability, the maximum yielding amount etc., which could provide basis for the selection of the yielding bolt. (3) With respect to coupling relationships between the yielding bolt and the surrounding rock, Guo et al. [15] introduced a new type of pressure-dispersive yielding bolt. The mechanism and mechanical behaviours were analysed, and the coupling relationships between

the yielding bolt and the surrounding rock were analysed and summarized; Zhang et al. [16] systematically analysed variations of surrounding rock stresses, displacements and axial forces before and after the installation of the yielding bolt. In term of the failure model, the yielding bolt could be subjected to two kinds of movement, either opening in the direction perpendicular to the plane or shearing in the plane [7, 17]. However, the failure of anchoring systems is mainly attributed to the shear loading. Indeed, many previous studies performed many experiments to study the factors that affect the shear behaviour of bolt, including bolt type. bolt diameter, bolt surface profile, bolt material, etc. [18-20].

Reviewer: 2

1. Adapt what exactly? Adapt is the incorrect choice of words in this context.

Thank you for your comments. The sentence is modified as follows:

In order to adapt to the high stress and avoid the large deformation of in roadways, the pre-stressed yielding bolt has been developed.

2. The logic does not follow on from previous sentence, that is “however” is incorrectly used here.

Thank you for your comments. The sentence is modified as follows:

Prior to the installation of the pre-stressed yielding bolt, boreholes need to be drilled. However, not all boreholes are perpendicular to the surface of the roadway, and the non-perpendicular holes make the pre-stressed yielding bolt exposed to eccentric loads.

3. What borehole? What connection is there between borehole and stress/deformation?

Thank you for your comments. The sentence is modified as follows:

Prior to the installation of the pre-stressed yielding bolt, boreholes need to be drilled. However, not all boreholes are perpendicular to the surface of the roadway, and the non-perpendicular holes make the pre-stressed yielding bolt exposed to eccentric loads.

4. This makes no sense

Thank you for your comments. The sentence is modified as follows:

However, not all boreholes are perpendicular to the surface of the roadway, and the non-perpendicular holes make the pre-stressed yielding bolt exposed to eccentric loads.

5. Why is this so?

Thank you for your comments. The sentence is modified as follows:

However, not all boreholes are perpendicular to the surface of the roadway, and the non-perpendicular holes make the pre-stressed yielding bolt exposed to eccentric loads.

6. I have not heard of these laws. Are the authors really intending on revealing the influence or determine/calculate etc?

Thank you for your comments. The sentence is modified as follows:

In order to reveal the influence laws of the eccentric load on the performance of the pre-stressed yielding bolt, ...

7. I think then authors mean "a simulation." There could/can be others

Thank you for your comments. The sentence is modified as follows:

In order to reveal the influence laws of the eccentric load on the performance of the pre-stressed yielding bolt, some numerical simulations were carried out in this paper study.

8. Were the simulations carried out in the paper or, are the results of the modelling and subsequent analysis presented in the paper?

Thank you for your comments. The simulations were carried out in the paper.

9. I am unfamiliar with this term, what is meant by this term?

Thank you for your comments. The term is changed to 'utilization rate for of the yielding pipe'.

10. Is the 'pipe material' yielding or, the 'yield pipe' is some sort of material that can have some sort of efficiency?

Thank you for your comments. The yielding pipe is one of components of the yielding pipe. The sentence is modified as follows:

The influence of the eccentric load on the displacement-load relations, utilization rate of the yielding pipe, the plastic strains of the bolt components, as well as the evolution of the absorptive capacity of the yielding pipe were analysed.

11. Again, I am unfamiliar with the term "use efficiency." What is it supposed to mean?

Thank you for your comments. The 'use efficiency' is modified as 'utilization rate'.

12. This is a statement rather than a conclusion...' to a certain degree' of what - relax, are near zero etc?

Eccentric loads affected the utilization efficiency of the yielding pipe material, plastic strains of bolt components, and the absorptive capacity was quite great when displacement was less than 2mm, while these impacts could be neglected when displacement is greater than 2mm.

13. What impacts? What 'later displacement?

Thank you for your comments. The sentence is modified as follows:

Eccentric loads affected the utilization rate of the yielding pipe, plastic strains of bolt components, and the absorptive capacity was quite great when displacement was less than 2mm, while these impacts could be neglected

when displacement is greater than 2mm.

14. What are "sound control effects?'

Thank you for your comments. The sentence is modified as follows:

Rock bolting support is one of the most common methods used in coal mines for its good controlling effect, low cost, and simple operation.

15. How is a something like a bolt which is an entity, an operation or process simple or otherwise?

Thank you for your comments. The sentence is modified as follows:

Rock bolting support is one of the most common methods used in coal mines for its good controlling effect, low cost, and simple operation.

16. What "bolt" are the authors referring to - some type of mechanical screw or, the specific type of support mechanism used in ground control of which there are two types - rockbolts and cable bolts. Are the authors referring to both these types of ground support bolts or to one or the other?

Thank you for your comments. The sentence is modified as follows:

Rock bolting support is one of the most common methods used in coal mines for its good controlling effect, low cost, and simple operation.

17. in the surrounding rockmass

Thank you for your comments. The sentence is modified as follows:

As the mining depth of coal mines increases, the roadways are subjected to a high stress and characterized with a large deformation.

18. Does the roadway have "high stress" or is the rockmass subjected to high stress?

Thank you for your comments. The sentence is modified as follows:

As the mining depth of coal mines increases, the roadways are subjected to a high stress and characterized with a large deformation.

19. What is "this time?" Or do the authors mean "Currently, common bolts…"

Thank you for your comments. The sentence is modified as follows:

Concerning the normal rock bolts cannot meet the support requirements in this condition, it is necessary to optimize their structures.

20. What are "common bolts?" How do they differ from say "rare" or "complex" bolts?

Thank you for your comments. The sentence is modified as follows:

Concerning the normal rock bolts cannot meet the support requirements in this condition, it is necessary to optimize their structures.

21. In what way are they "difficult?" Do the author mean they are unable to meet the service requirements need for ground control?

Thank you for your comments. The sentence is modified as follows:

Concerning the normal rock bolts cannot meet the support requirements in this condition, it is necessary to optimize their structures.

22. I am unfamiliar with what and how "the structures" have been "optimised".

Thank you for your comments. Structural optimization refers to adding yielding pipe to normal rock bolts.

23. What are "extensible bolts?"

Thank you for your comments. We deleted that term.

24. Background information about bolt should be referenced by the authors.

Thank you for your comments. The background information about bolt added some references.

[4] Nassar, S.A. & Matin, P.H. 2007 Cumulative clamp load loss due to a fully reversed cyclic service load acting on an initially yielded bolted joint system. Journal of Mechanical Design 129, 421-433. (doi:10.1115/1.2429700).

[5] Ansell, A. 2005 Laboratory testing of a new type of energy absorbing rock bolt. Tunnelling and Underground Space Technology 20, 291-300. (doi: 10.1016/j.tust.2004.12.001).

[6] Mohammadi, M., Hossaini, M.F. & Bagloo, H. 2015 Rock bolt supporting factor: rock bolting capability of rock mass. Bulletin of Engineering Geology and the Environment 76, 231-239. (doi:10.1007/s10064-015-0785-y).

[7] Grasselli, G. 2005 3D Behaviour of bolted rock joints: experimental and numerical study. International Journal of Rock Mechanics and Mining Sciences 42, 13-24. (doi: 10.1016/j.ijrmms.2004.06.003).

25. Can

Thank you for your comments. The sentence is modified as follows:

It can be seen that the yielding bolt consists of a nut, yielding pipe, tray and high-intensity bolt body.

26. A nut

Thank you for your comments. The sentence is modified as follows:

It can be seen that the yielding bolt consists of a nut, yielding pipe, tray and high-intensity bolt body.

27. "rod body" does not appear in Figure 1a. Do the authors mean "bolt body?"

Thank you for your comments. The sentence is modified as follows:

It can be seen that the yielding bolt consists of a nut, yielding pipe, tray and high-intensity bolt body.

28. Why is the "yielding pipe" more of a core component compared to the other components? What is core and which are non-core?

Thank you for your comments. The sentence is modified as follows:

As a component for yielding, the yielding pipe is made of seamless steel pipe.

29. What is "deformation energy?' What is its source? Under what circumstances does it become "excessive?"

Thank you for your comments. The energy released in the process of roadway deformation is called deformation energy. When the roadway deformation exceeds 5% ~ 10% of the bolt length, the bolt breaks, and this deformation is considered to be excessive.

30. What are "constraint forces?' Why does yielding lead to higher constrain forces?"

The constraint forces refer to a kind of squeezing force produced by anchor bolt on the roadway. When further increase of the roadway deformation after yielding, the deformation of the bolt increases, and the squeezing force of the bolt increases, which leads to greater constraint force.

31. How can "constraint forces" lead to effective control or an underground roadway?

Thank you for your comments. The rock bolt does not have a yielding function and the its deformation is small. When the roadway deformation is large, the rock bolt breaks and loses its constraint for the roadway. However, the yielding bolt have the yielding function and could happen to be large deformation. Due to the release of a large amount of deformation energy in the roadway deformation, the deformation in the later period will be reduced, which will reduce the probability of fracture of the yielding bolt. After large deformation of the roadway, they yielding bolt still has a constraint force for roadway, which is more conducive to an underground roadway.

32. I cannot appreciate how yielding of a bolt leads to better stability

Thank you for your comments. The rock bolt does not have a yielding function and the its deformation is small. When the roadway deformation is large, the rock bolt breaks and loses its constraint for the roadway. However, the yielding bolt have the yielding function and could happen to be large deformation. Due to the release of a large amount of deformation energy in the roadway deformation, the deformation in the later period will be reduced, which will reduce the probability of fracture of the yielding bolt. After large deformation of the roadway, they yielding bolt still has a constraint force for roadway, which leads to better stability.

33. Is required

Thank you for your comments. Before installation of the yielding bolt, drilling equipment is required to drill holes.

34. of a given diameter, to a certain prescribed depth and at designed inclination.

Because there are many types of yielding bolt and the drilling depth is related to the engineering geological conditions, it is difficult to give the above parameters. Therefore, the sentence is modified as follows:

Before installation of the yielding bolt, drilling equipment is required to drill holes.

35. Professional as opposed to non-professional?

Thank you for your comments. The sentence is modified as follows:

Before installation of the yielding bolt, drilling equipment is used to drill holes.

36. Isn’t (in)accuracy an outcome rather than a determining factor? What are “personal operations?” I think there are other factors also that result in deviation from the designed drill pattern.

Thank you for your comments. The sentence is modified as follows:

Due to designed drill pattern, equipment accuracy, and personal misoperation, borehole deviation usually takes place during the drilling process, as shown in Figure 2.

37. What does this mean?

Thank you for your comments. Due to designed drill pattern, equipment accuracy, and personal misoperation, borehole deviation usually takes place during the drilling process, as shown in Figure 2. Consequently, the eccentric load will happen to the pre-stressed yielding bolt.

38. Where exactly is “home” and “abroad?” It is relative - the authors backyard, neighbourhood, province, region or do they mean country?

Thank you for your comments. In-depth studies have been made on the yielding bolt comprehensively, mainly focusing on evaluation of the yielding effects, selection of key parameters of the yielding bolt, and coupling relationships between the yielding bolt and the surrounding rock.

39. With respect to the evaluation of the yielding effect, Xiang et al [5]...

Thank you for your comments. The sentence is modified as follows:

With respect to the evaluation of the yielding effect, Xiang et al. [1] established a mechanical model of the yielding bolt. The finite element shear strength reduction method was used to analyse and evaluate the support effects of the high-intensity yielding bolt;

Appendix D

Cover letter

Dear editor

Thank you for giving me the chance to revise my manuscript. A native English speaker, a PhD. from the University of Hawaii at Manoa, polished the language of my text. Besides, a description of bolts has been added into the text. All revisions are marked in MS track mode. If you have any questions, please contact me.

Yours sincerely,

Yang Tai
E-mail: cumtcqty@163.com

The Revision for Comments

Associate Editor Comments

1. change "Mises stress" to "von Mises stress"

Thanks for your comments. The "Mises stress" has been changed to "von Mises stress".

2. units in tables and graphs should be provided in (), i.e. (MPa) and not /MPa. Please consult the English literature on how units are represented

The form of units has been changed.

3. The inner and outer diameter of the yielding pipe are not given. Table 2 provides 20 and 22 mm for the yielding bolt, and provides the yielding pipe height but not the inner and outer diameters.

Thanks for your comments. The inner and outer diameters yielding pipe are 25mm and 40.5mm, respectively.

4. What does "Materials" in Table 2 correspond to? Is that the yield strength of the steel? If so, adjust the heading in the table accordingly.

Thanks for your comments. The "Materials" has been changed to the "Materials of yielding pipe"

5. bolt diameter is a better expression compared to bolt's diameter (both are in the text)

Thanks for your comments. The bolt's diameter has been changed to the bolt diameter.

6. tray in figure 6 should be plate. Consult the international literature

The tray has been changed to plate.

7. "All main elements surface" is not a good expression, please rephrase

The "All main elements surface" has been changed to the master surface.
